# Complement dysregulation during the early phases of synucleinopathy

**DOI:** 10.1007/s00401-026-03057-8

**Published:** 2026-07-20

**Authors:** Hina Khan, Mary Gifford, Arash Kordbacheh, Asher Bury, Spencer Panoushek, Allyson Cole-Strauss, Christopher J. Kemp, Kelvin C. Luk, Kathy Steece-Collier, Nathan C. Kuhn, Nicholas M. Kanaan, Caryl E. Sortwell, Joseph R. Patterson, Matthew J. Benskey

**Affiliations:** 1 Department of Translational Neuroscience, Michigan State University, Grand Rapids, MI 49503 USA; 2 Cell and Molecular Biology Program, Grand Valley State University, Allendale, MI USA; 3 Biomedical Sciences Program, Grand Valley State University, Allendale, MI USA; 4 Department of Pathology and Laboratory Medicine, Center for Neurodegenerative Disease Research, University of Pennsylvania Perelman School of Medicine, Philadelphia, PA USA

**Keywords:** Complement system, Alpha-synuclein, Synucleinopathy, Neuroinflammation, Parkinson’s disease, Neurodegeneration

## Abstract

Parkinson’s disease (PD) is characterized by progressive degeneration of nigrostriatal dopamine neurons and synucleinopathy, which is the accumulation of aggregated α-synuclein (α-syn). Increasing evidence implicates α-syn-associated neuroinflammation as a contributor to PD pathogenesis, yet immune mechanisms linking synucleinopathy to neurodegeneration remain incompletely defined. Activation of the complement cascade occurs in PD and other synucleinopathies, but most studies report complement activation after overt neurodegeneration, making it difficult to conclude if complement is directly activated by pathological α-syn or secondarily following neurodegeneration. We used the rat α-syn preformed fibril (PFF) model, in vitro complement assays and postmortem human PD tissue to investigate whether pathological α-syn directly activates complement prior to overt neurodegeneration. The α-syn PFF model exhibits a protracted pathological time course and distinct temporal separation between peak α-syn aggregation and nigrostriatal degeneration. Thus, we quantified complement expression, activation, and regulation during the aggregation phase. Synucleinopathy caused complement activation prior to nigrostriatal degeneration, including upregulation of components of both the classical (*C1qa, C1r, C4b*) and alternative (*Cfd, Cfb*) pathways, the anaphylatoxin (*C3aR, C5aR*) and phagocytic (*CR3*) complement receptors, and activation of complement C3. During early synucleinopathy microglia upregulated C3, which significantly correlated with synucleinopathy burden across several brain regions, including the substantia nigra pars compacta (SNc) and cortex. Concurrently, complement regulators, including Cd55, Cd59, neuronal pentraxin-1 (Nptx1), and the neuronal pentraxin receptor were downregulated in the synucleinopathy-affected SNc. Importantly, increased levels of C1q and iC3b along with downregulation of CD55 and NPTX1 protein were also observed in human postmortem PD SNc tissue, supporting the translational relevance of our findings. Mechanistically, we demonstrate that aggregated, but not monomeric, α-syn directly binds C1q and activates the complement cascade in a C1q-dependent manner. These data provide the first in vivo evidence that synucleinopathy triggers complement activation and dysregulation prior to neurodegeneration.

**Supplementary Information:**

The online version contains supplementary material available at 10.1007/s00401-026-03057-8.

## Background

Parkinson’s disease (PD) is a progressive neurodegenerative disorder associated with clinical symptoms of parkinsonism (bradykinesia, resting tremor and postural instability) and a variable constellation of non-motor symptoms. Neuropathologically, PD is characterized by the degeneration of nigrostriatal dopamine neurons and accumulation of aggregated forms of the protein α-synuclein (α-syn) [10, 72]. Neuroinflammation is widely recognized as a central feature of PD pathology, involving chronic and systemic activation of both innate and adaptive immunity (reviewed in [34]. Importantly, indices of immune activation are present in prodromal and de novo PD patients and correlate with the degree of clinical symptomology and/or the time of symptom onset [14, 16, 31, 38, 39, 53, 69, 73, 74, 89, 92], suggesting immune activation is an active participant in disease progression. Collectively these data implicate neuroinflammation in PD pathogenesis, yet the exact mechanism(s) by which neuroinflammation drives neurodegeneration remain incompletely defined. As such, deciphering mechanisms of early-stage immune activation in PD may shed light on disease pathogenesis.

Synucleinopathy is likely a primary driver of the immune activation in PD. In the PD brain, activated microglia spatially and temporally correlate with Lewy pathology [7, 27], antibodies and lymphocytes targeting α-syn are present in PD patients [52, 70, 77], and microglia and monocyte activation are characteristic features of α-syn-based models of PD [12, 22, 23, 65, 68, 79, 85]. Based on this, we sought to identify early-stage immune mediators that respond directly to synucleinopathy. The complement system is a division of the innate immune system that continuously surveils the body and acts as a first responder to a wide range of immunogenic stimuli, including pathogens and cellular damage. As such, we hypothesized complement activation represents a fundamental early-stage driver of immune activation in response to pathological α-syn.

**Fig. 1:**
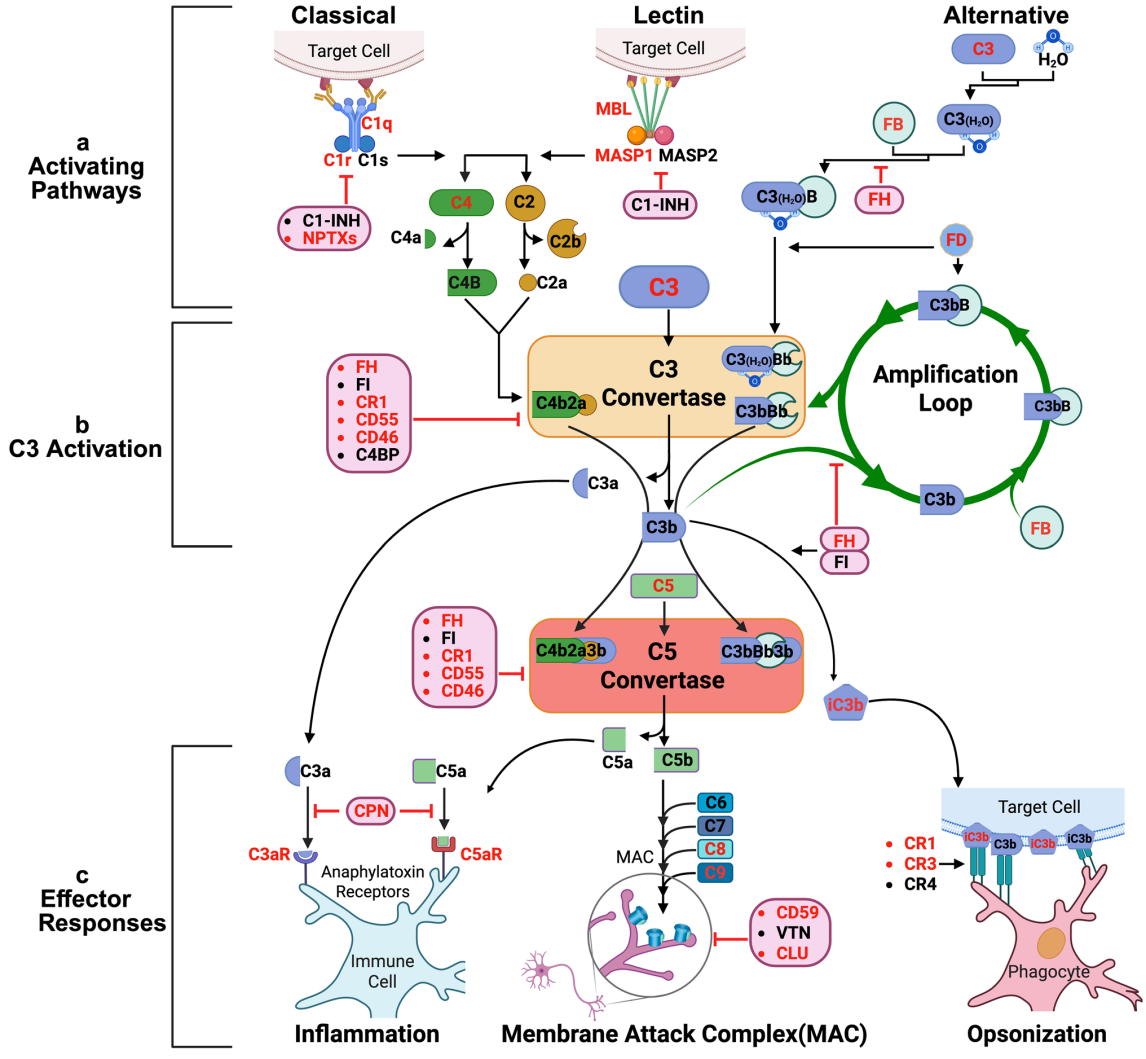
The complement cascade. The complement system is an arm of innate immunity, comprising ~ 50 proteins ubiquitously present in tissues and activated by proteolytic cleavage. **a** Complement activation occurs via one of three pathways: Classical, lectin, and alternative. The classical pathway is initiated when C1q binds targets, activating C1r and C1s, which cleave C4 and C2 to form the C3 convertase, C4b2a. The lectin pathway is triggered by mannose-binding lectin (*MBL*), collectins, or ficolins recognizing microbial- or altered self-carbohydrates, activating MASP-1 and MASP-2 to generate the C3 convertase, C4b2a. The alternative pathway is constitutively active through spontaneous C3 hydrolysis, forming the free C3 convertase C3(H2O)Bb, and is amplified on surfaces to form the alternative pathways C3 convertase, C3bBb, enhancing complement activation. **b** The central event in the cascade is cleavage of C3 by the C3 convertases to form C3a and C3b, which initiate the major effector responses of the system. **c** Effector Responses: Opsonization/Phagocytosis: C3b and iC3b deposited on target surfaces promote phagocytosis via CR1, CR3, and CR4. Inflammatory Signaling: The anaphylatoxin peptides, C3a and C5a, mediate inflammatory signaling through C3aR and C5aR, driving chemotaxis, cytokine production, and activation of immune cells. Membrane Attack Complex (*MAC*): C5b initiates the terminal pathway by recruiting C6–C9 to form the MAC, lysing target cells. Complement activity is tightly regulated at multiple levels (shown in pink boxes): C1 inhibitor (*C1-INH*) and neuronal pentraxins (*NPTXs*) restrain classical initiation, C4 binding protein (*C4BP*), Factor H (*FH*), and FI regulate C3 convertases and alternative pathway amplification; carboxypeptidase-N (*CPN*) inactivates anaphylatoxins; the membrane bound regulators CR1 (AKA CD35), CD46, CD55, and CD59 prevent deposition of activated complement on self-cells; clusterin (*CLU*) and vitronectin (*VTN*) prevent MAC assembly and insertion. Representative complement components (shown in red text) were quantified in α-synuclein pre-formed fibril–injected rats and postmortem PD brains

The complement system consists of ~ 50 proteins that circulate through the body as inactive precursors (Fig. 1). Activation of the complement system is initiated via one of three pathways: The classical, lectin, and alternative pathways (Fig. 1a). All pathways result in the sequential proteolytic cleavage of complement proteins that ultimately converge on the cleavage-mediated activation of complement component 3 (C3; Fig. 1b). Activation of C3 initiates the main effector responses of the complement cascade (Fig. 1c), including 1) the deposition of opsonins (C3b and iC3b) on targets to tag them for phagocytic clearance, 2) production of the anaphylatoxins, C3a and C5a, that recruit and activate immune cells, and 3) the lytic destruction of cells following insertion of the membrane attack complex (MAC) in target membranes. Complement proteins are ubiquitously present, rapidly activated, and coordinate inflammation to a wide range of immunogens, making complement uniquely situated to act as a first responder to synucleinopathy.

Existing data document complement activation in PD. For instance, complement expression is increased in the substantia nigra pars compacta (SNc) and caudate of PD patients [30, 46], while complement proteins are altered in the CSF and blood of PD patients, with some changes predicting PD up to 7 years prior to motor symptom onset [17, 18, 21, 35, 48, 50, 82, 86]. Notably, complement activation is enriched in synucleinopathy-affected regions, where neuromelanin neurons and Lewy bodies label with opsonins and components of the MAC in the PD brain [9, 42, 87], and Lewy bodies label with activated complement in Dementia with Lewy body (DLB) [28, 81]. Similarly, complement is activated in preclinical α-syn-based models of PD [6, 43, 55, 75, 88, 94]. Collectively, these data strongly imply pathological α-syn drives complement activation in PD and other synucleinopathies. However, complement activation in PD or associated animal models has been assessed after significant cell death has occurred [6, 9, 42, 43, 87, 94]. Thus, a key unresolved question is whether complement is directly activated by pathological α-syn prior to cell death, where it may drive neurodegeneration.

We recently reported that SNc glia rapidly upregulate expression of complement genes months prior to degeneration in the rat α-syn preformed fibril (PFF) model of synucleinopathy [57, 75]. We have identified two distinct pathological phases that occur in the SNc following intra-striatal injection of α-syn PFFs in rats [11, 12, 56, 59, 76]. The first phase or “aggregation phase” occurs 1–2 months post-injection and is characterized by peak accumulation of “Lewy body-like” α-syn aggregates in nigral neurons. The second phase, or “degenerative phase” occurs 4–6 months post-injection and is characterized by progressive degeneration of nigrostriatal dopamine neurons. Importantly, complement *C3* and *C1q* are significantly upregulated in SNc glia during the aggregation phase, 2–4 months prior to overt neurodegeneration [75]. Thus, the purpose of the current study is to provide an in-depth investigation of complement activation during the early stages of synucleinopathy and determine if pathological α-syn directly activates complement prior to overt neurodegeneration.

To accomplish this, we leveraged the protracted time course of pathology in the rat α-syn PFF model to quantify complement expression and activation during the aggregation phase (targets shown in red text in Fig. 1). We report that induction of synucleinopathy results in robust complement activation prior to overt neurodegeneration, including (1) upregulation of genes in the classical and alternative complement pathways, (2) upregulation and activation of C3 that correlates with synucleinopathy burden in the SNc and cortex (Cx), and (3) a significant downregulation of specific complement regulators in the SNc. We validated downregulation of CD55 and NPTX1 in human postmortem PD SNc tissue and demonstrate that aggregated α-syn, but not monomeric α-syn, directly binds C1q and activate the classical complement system in a C1q-dependent manner. These results demonstrate robust complement activation and dysregulation during the early stages of synucleinopathy (i.e. prior to cell death), confirming the ability of pathological α-syn to directly activate the complement system.

## Materials and methods

### Animals

Three-month-old, male and female Fischer 344 rats (CDF, strain code 002) were purchased from Charles River Laboratories and housed 2–3 per cage at the Michigan State University Grand Rapids Research Center, which is fully approved through the Association for Assessment and Accreditation of Laboratory Animal Care (AAALAC). Rats were housed in a room with a 12-h light/dark cycle and provided food and water ad libitum. All procedures were performed in accordance with federal, state and institutional guidelines approved by the Michigan State University Institutional Animal Care and Use Committee (IACUC).

### Postmortem human tissue

Age matched, fresh frozen and formalin-fixed paraffin-embedded postmortem midbrain tissue from controls and individuals with a neuropathological diagnosis of PD was obtained from the Michigan Brain Bank (See supplemental Table 1 online resource for detailed neuropathological and demographic data). Neuropathologic diagnoses were obtained from de-identified autopsy reports generated by board-certified neuropathologists. Detailed pathologic features were extracted from the reports, including descriptions of SNc degeneration, Lewy body pathology, and co-occurring neuropathologies. Lewy pathology distribution was categorized using standard criteria (brainstem-predominant, limbic/transitional, or diffuse neocortical) when supported by report text. Alzheimer disease neuropathologic change was classified according to NIA-AA guidelines when Braak stage and/or CERAD plaque scores were available. Features not explicitly reported were left unassigned. For biochemical analysis of postmortem tissue, fresh frozen midbrain tissue was placed on a cooling stage and viewed under a dissecting microscope. Tissue punches from the SNc were obtained with a round, 2 mm diameter tissue punch inserted 2 mm (2 mm x 2 mm tissue punch) and frozen at -80 °C until further processing.

### Production of recombinant α-syn monomers and PFFs

To produce recombinant mouse α-syn protein, a plasmid encoding wild type (WT) mouse α-syn (PRK172, kindly gifted by Luk lab) was transformed into BL21 (DE3) RIL *E. coli* (ThermoFisher C600003) and cultured overnight at 37 °C in Terrific Broth (TB) containing carbenicillin (100 μg/mL) with shaking at 250 RPM. For recombinant human α-syn protein, a plasmid encoding WT human α-syn (Addgene Plasmid #36,046) was transformed into One Shot BL21 (DE3) Star *E. coli* (ThermoFisher, C601003) and cultured at 37 °C in TB containing carbenicillin (100 μg/mL) with shaking at 250 RPM. When cultures reached an optical density of 0.6 at A600, protein expression was induced by adding isopropyl β-D-1-thiogalactopyranoside (IPTG) to a final concentration of 1 mM, and cultures were grown an additional three hours.

Cultures were centrifuged at 5300 × g for 20 min at 4 °C, supernatant decanted, and pellets collected. Pellets were resuspended in ice-cold High Salt Buffer (750 mM NaCl, 10 mM Tris, 1 mM EDTA, 1 mM PMSF, with protease inhibitor cocktail tablet (Sigma P8340)). Pellets were homogenized by three rounds of sonication using a Qsonica Q125 Sonicator connected to a 20 mm horn set at amplitude 70%, with 30 s pulses and 30 s rest between pulses. The homogenate was boiled for 20 min, chilled on ice, and centrifuged at 5300 × g for 20 min at 4 °C. Supernatant was dialyzed overnight at 4 °C in Buffer A (10 mM Tris pH 7.6, 1 mM EDTA). Retentate was then centrifuged 5300 × g for 20 min at 4 °C and filtered with a 0.45 µm PVDF filter.

α-syn protein was purified with two rounds of ion exchange FPLC (Cytiva AKTA Pure 25; Unicorn 7.6). Protein was loaded onto a HiPrep Q FF 16/10 column and eluted against a linear gradient of 7 column volumes of Buffer B (10 mM Tris pH 7.6, 1 mM EDTA, 1 M NaCl). Collected fractions were run on an AnyKD Criterion TGX gel (BioRad, 5,671,124) and stained with Coomassie blue (ThermoFisher 20,279). Fractions containing a band the size of α-syn (~ 14 kDa) were dialyzed overnight at 4 °C in Buffer A. Protein was then loaded onto a HiTrap Q HP column and eluted against a linear gradient of 9 column volumes of Buffer B (10 mM Tris pH 7.6, 1 mM EDTA, 1 M NaCl). Collected fractions were run on an AnyKD Criterion TGX gel as before and fractions with a single band at ~ 14 kDa were dialyzed overnight at 4 °C in Buffer A. Endotoxin removal was performed with Pierce High-Capacity Endotoxin Removal Spin Columns (ThermoFisher 88,274) per manufacturer instructions, then dialyzed overnight at 4 °C in 10 mM Tris pH 7.6 containing 50 mM NaCl. Retentate was centrifuged at 10,000 × g for 30 min at 4 °C, protein content quantified by A280 on a NanoDrop 8000 spectrophotometer (ThermoFisher) and diluted to a concentration of 5 mg/mL. A portion of purified α-syn was flash-frozen in ethanol supercooled on dry ice and stored at −80 °C as α-syn monomer control. The remaining recombinant α-syn was used to generate PFFs. For fibrilization, 300 µL aliquots of recombinant α-syn monomers (5 mg/mL) were shaken at 1,000 RPM for 7 days at 37 °C[58]. Fibrils were aliquoted, flash-frozen in ethanol supercooled on dry ice chilled and stored at −80 °C. Endotoxin contamination was tested using the *Limulus* amebocyte lysate assay and all PFF preparations contained < 0.5 endotoxin units/mg of total protein. Both monomer and fibrils underwent in vitro testing (described below) and in vivo testing in rodents to ensure seeding efficacy before use in experiments.

### Recombinant α-syn PFF in vitro validation and sonication

Recombinant α-syn PFF amyloid structure, oligomerization, and length were confirmed in vitro using Thioflavin-T (ThT) fluorescence, sedimentation assay, and transmission electron microscopy, respectively (Supplemental Fig. 1 online resource). Amyloid structures in recombinant full-length PFFs were confirmed using a ThT assay as described [58]. Briefly, α-syn monomers and PFFs were diluted in Dulbecco’s phosphate buffered saline (dPBS) to a concentration of 0.1 mg/mL and incubated with ThT in glycine buffer (25 μM ThT, 100 mM glycine, 1% Triton X-100, pH 8.5) for 1 h at room temperature (RT). ThT fluorescence was quantified using a BioTek Synergy H1 plate reader (Agilent) with excitation of 450 nm and emission of 510 nm (Supplemental Fig. 1a, b online resource). α-syn oligomerization was confirmed using a sedimentation assay as described [58]. Briefly, α-syn monomers and PFFs were diluted in dPBS to a concentration of 0.5 mg/mL and centrifuged at 10,000 × g for 30 min at RT, after which supernatants were transferred to a new tube. Protein in the supernatant and pellet fraction were separated by SDS-PAGE using AnyKD Criterion TGX gel (BioRad, 5,671,124). Gels were then stained with Coomassie blue (ThermoFisher 20,279) and α-syn resolving in the supernatant and pellet fractions from monomer and PFF preparations were validated (Supplemental Fig. 1c, d online resource).

Prior to stereotactic injection, mouse α-syn PFFs were thawed to RT and diluted to 4 mg/mL in sterile dPBS and sonicated with a Qsonica Q700 Cuphorn sonicator connected to a thermocube cooler set to 15 °C. PFFs were sonicated at amplitude 30 with cycles of 3 s of sonication followed by a 2 s pause, for a total of 15 min of sonication. Samples were removed and gently triturated to mix the solution and then sonicated at amplitude 30 for another 7.5 min, again with cycles of 3 s of sonication followed by a 2 s pause at 15 °C.

Optimal size of sonicated mouse α-syn PFFs was confirmed using transmission electron microscopy (EM; Supplemental Fig. 1e–g online resource). For EM of PFFs, Formvar/carbon-coated copper grids (EMSDIASUM, FCF300-Cu) were washed twice with ddH2O, then sonicated fibrils were diluted 1:50 in sterile dPBS and 10ul of diluted PFFs were adsorbed on grids for 1 min, followed by a 1-min incubation in 10 µl of aqueous 2% uranyl acetate (Electron Microscopy Sciences 22,400). Grids were allowed to dry fully before being imaged with a JEOL JEM-1400 + transmission electron microscope. Fibril lengths were measured from > 500 individual fibrils using ImageJ [67] to ensure samples contained PFFs with fibril length ≤ 50 nm, which is required for successful cellular uptake and seeding of endogenous α-syn [78]. The mean length of sonicated PFFs was 36 ± 14 nm (Supplemental Fig. 1e–g online resource).

### Surgical cohorts and stereotaxic injections

For the current study, three surgical cohorts were used to generate tissue for ddPCR, biochemical and histological analyses, respectively. Each cohort consisted of male and female rats that were randomly assigned to receive injections of either mouse α-syn PFFs or PBS vehicle control. Additionally, the surgical cohort used for biochemistry included male and female rats that received injections of mouse α-syn monomer control protein. The same lot of α-syn PFFs was used for all surgical cohorts. Surgical cohort 1 (*n* = 6–8/group/sex) was used for ddPCR based gene expression analyses, surgical cohort 2 (*n* = 4–5/group/sex) for biochemical analyses, and surgical cohort 3 (*n* = 5–7/group of mixed sexes) for immunofluorescent analyses. Intra-striatal injection of sonicated mouse α-syn or dPBS vehicle control were conducted as previously described [56, 75]. Rats were anesthetized with isoflurane (5% induction and 1.5% maintenance) and received unilateral intra-striatal injections to the left hemisphere (2 injections of 2 µl each). Site 1: AP + 1.0, ML + 2.0, DV −4.0; Site 2: AP + 0.1, ML + 4.2, DV −5.0. All AP and ML coordinates are relative to Bregma, while DV coordinates are relative to dura. Recombinant mouse α-syn PFFs or mouse α-syn monomer protein (4 mg/mL; 2 × 2 μl injection; 16 ug total) or an equal volume of dPBS vehicle were injected at a rate of 0.5 µl/minute with a pulled glass capillary tube attached to a 10 µl Hamilton syringe. After each injection the needle was left in place for 1 min, retracted 0.5 mm then left for another 2 min before fully retracting to prevent reflux. All animals received analgesic (1.2 mg/kg of sustained release buprenorphine; Ethiqa XR) and were monitored daily for 4 days post-surgery to ensure full recovery.

### Euthanasia and tissue preparation

Animals were euthanized at 2 months (60 days) post-injection using an injection of pentobarbital (30 mg/kg i.p.; Euthanasia-III Solution, Med-Pharmex Incorporated) and perfused intracardially with heparinized (10,000 units/L) 0.9% saline. For ddPCR and protein biochemistry, brains were removed and immediately flash frozen in 2-methylbutane supercooled on dry ice for 10–20 s. Frozen brains were stored at − 80 °C until further processing. Regions of interest (SN and ST) were microdissected from frozen brains using a modified version of the Palkovits punch technique [54]. Briefly, frozen brains were mounted in OTC (Fisher Healthcare Tissue-Plus OCT) and sectioned on a 305S cryostat (Leica Biosystems) set at − 15 °C to the level of the rostral ST (~ 1.96 mm posterior from bregma). The ST was microdissected using a 2 mm round biopsy punch, inserted to a depth of 2 mm (2 mm x 2 mm tissue punch). Brains were further sectioned to the level of the rostral SN (~ 4.5 mm posterior from bregma), and the SN (including the SNc) was micro-dissected using a custom biopsy punch (0.5 mm × 1.5 mm), inserted to a depth of 2 mm (~ 1 mm x 2 mm tissue punch). Tissue for ddPCR was collected in DNase/RNase free microcentrifuge tubes containing 100 µl TRIzol reagent (Invitrogen 26,696,026), homogenized with a disposable pestle, the volume of TRIzol was brought to 1 mL, triturated until homogenous, and samples were frozen on dry ice and stored at − 80 °C. Tissue used for biochemistry was collected in microcentrifuge tubes and immediately frozen on dry ice then stored at − 80 °C. For histology, animals were perfused with 4% paraformaldehyde following saline perfusion. Brains were removed and post-fixed in 4% paraformaldehyde overnight, then cryopreserved in 15% sucrose in 0.1 M phosphate buffer until saturated, followed by 30% sucrose in 0.1 M phosphate buffer until saturated. Brains were maintained in 30% sucrose solution until sectioning. To section, brains were frozen with dry ice on the platform of a freezing stage, sliding knife microtome and sectioned coronally at 40 µm into 6 series of sections spanning the entire rostral-caudal axis of the brain. Sections were stored in 30% sucrose, 30% ethylene glycol, in 0.1 M phosphate buffer, pH 7.3 at 4 °C.

### RNA isolation

RNA isolation was conducted as described previously [75]. Tissue punches in TRIzol were thawed on ice, briefly centrifuged then transferred to Phasemaker tubes (ThermoFisher A33248) and incubated at RT for 5 min. Chloroform (200 µl/sample) was added and tubes were mixed thoroughly by hand then incubated at RT for 10 min, followed by centrifugation at 16,000 × g at 4 °C for 5 min. Following centrifugation, the aqueous phase was transferred to a clean RNase free tube, and an equal volume of 100% ethanol was added to tubes and vortexed well. RNA was purified using an RNA clean and concentrator kit (Zymo Research, R1016) according to the manufacturer’s instructions with slight modification. Sample was added to the column, centrifuged for 1 min at 12,000 × g, then columns were washed with 400 µl of RNA wash buffer and centrifuged for 1 min at 12,000 × g. DNA was removed by incubating column membranes with 3 Units of DNase I (Thermo Scientific EN0521) at RT for 15. Columns were centrifuged at 12,000 × g for 1 min to remove DNase, then 400 µl of RNA prep buffer was added and columns were centrifuged at 12,000 × g for 1 min. Columns were washed with 700 µl RNA wash buffer, centrifuged at 12,000 × g for 1 min, washed with another 400 µl of RNA wash buffer and centrifuged at 12,000 × g for 1 min. A final centrifugation was done at 12,000 × g for 3 min and columns were allowed to air dry at RT for 5 min to remove residual wash buffer. DNase/RNase free water (30 µl/sample) was added to the column and incubated for 5 min then eluted by centrifugation at 10,000 × g for 1 min. Quality and quantity of RNA was assessed with Agilent 2100 Bioanalyzer using an Agilent RNA 6000 Pico Kit (5067–1513). RNA was diluted to 1 ng/µL with DNase/RNase free water, aliquoted and stored at − 80 °C.

#### ddPCR

ddPCR was performed as previously described [75]. cDNA was prepared from 10 ng of purified RNA using iScript Reverse Transcription Supermix (Bio-Rad, 1,708,841) according to manufacturer’s instructions, then diluted 1:1 with cDNA storage buffer (10 mM Tris HCl pH 7.5, 0.1 mM EDTA pH 8.0) and stored at − 20 °C. TaqMan Gene Expression Probes (Applied Biosystems) were used for ddPCR. All respective probes (See Supplemental Table 2 online resource for probe details) were species specific and spanned exon-exon junctions whenever possible. For ddPCR reactions, a mastermix was made by combining 9 μl of ddPCR supermix (BioRad 1,863,024) with 1 ul of probe of interest conjugated to FAM fluorophore, and 1 ul of *Rpl13* probe conjugated to VIC fluorophore. Mastermix was combined with 11 μl of cDNA and 20 µL of the cDNA-master mix reaction was added to a DG8 droplet generator cartridge (Bio-Rad, 1,864,008) with 70 µL of droplet generation oil (Bio-Rad, 1,863,005). A QX droplet generator (Bio-Rad, 186–4002) was used to produce droplets containing the cDNA/mastermix reaction. Droplets (40 μl) were transferred to a 96-well plate (Bio-Rad, 12,001,925) and sealed with pierceable foil (Bio-Rad, 181–4040) by a plate sealer (Bio-Rad, 181–4000). Plates were transferred to a thermocycler (Bio-Rad, C1000), and the PCR reaction was carried out with the following settings: 10 min at 95 °C, 39 cycles (30 s at 94 °C, 1 min at 60 °C), 10 min at 98 °C, hold at 12 °C (constant lid temperature of 105 °C). Plates were then transferred to the QX200 droplet reader (Bio-Rad, 1,864,003), and results analyzed with QuantaSoft software. For all samples, the gene of interest was normalized to the reference gene, *Rpl13*, and final data is expressed as fold change from PBS controls.

### SDS-page and western blotting

Tissue punches from the rat ST and SN, and human SNc, were homogenized in 10 volumes (w/v) of RIPA buffer (50 mM Tris pH 7.4, 150 mM NaCl, 1% NP-40, 0.5% sodium deoxycholate, 1% sodium dodecyl sulfate (SDS), 1 mM Ethylenediaminetetraacetic acid (EDTA)) containing protease (10 μg/mL pepstatin, leupeptin, bestatin and aprotinin, and 1 mM Phenylmethylsulfonyl fluoride (PMSF)) and phosphatase inhibitors (1 mM tetra-sodium pyrophosphate decahydrate, 10 mM beta-glycerophosphate disodium salt pentahydrate, 1 mM sodium orthovanadate, 10 mM sodium fluoride) by sonication using 10 short pulses at power 2 (Misonix XL-200). Crude lysates were centrifuged at 13,000 × g for 20 min at 4° C and the supernatant containing soluble protein was saved. Protein content was determined using a Pierce rapid gold BCA kit (Thermo, A53225) according to the manufacturer’s instructions. To test the specificity of the phospho-serine 129 α-syn (pSyn) antibody used for biochemical quantification of pSyn, lysates were incubated overnight at 37 °C with and without FastAP Thermosensitive Alkaline Phosphatase (7.5U; ThermoFisher EF0651). For all samples, 25–60 μg of protein was mixed with 6 × Laemmli sample buffer and heated to 98 °C for 5 min. Proteins were separated by SDS-PAGE using AnyKD Criterion TGX gels (BioRad, 5,671,124) and transferred to BioTrace nitrocellulose membranes (VWR, 27,376–991). Membranes were blocked in tris buffered saline (TBS; 0.15 M sodium chloride, 0.05 M Tris, pH7.6) containing 2% non-fat dry milk (Sanalac) for 1 h prior to overnight incubation at 4 °C in primary antibodies. Primary antibodies and the concentrations used are listed in Supplemental 3 online resource. Membranes were then washed 3 × 5 min in tris-buffered saline (TBS) containing 0.1% tween-20 (TBS-T). For immunoblotting of rat C3 only, membranes were incubated in a goat anti-rabbit horseradish peroxidase conjugated secondary antibody (Jackson ImmunoResearch 111-035-144; RRID:AB_2307391) diluted at 1:5,000 for 2 h at RT, then developed with an enhanced chemiluminescent substrate (BioRad 1,705,062) according to manufacturer’s instructions. Rat C3 membranes were washed 3 × 5 min in TBS before being imaged on a Biorad Chemidoc Imager. For all other target proteins, membranes were incubated in either goat anti-mouse IRDye 800CW (LiCor 926–32,210; RRID: AB_621842), goat anti-rabbit IRDye 680RD (LiCor 926-68,071; RRID: AB_2313606), or donkey anti-goat IRDye 680RD (LiCor 926–68,074; RRID: AB_10956736) diluted at 1:20,000 for 2 h at RT. Membranes were washed 3 × 5 min in TBS-T and then imaged on a LiCor Odyssey near-infrared imaging system with ImageStudio software (v5.2.5, LiCor Biosciences).

### Sandwich enzyme linked immunosorbent assay (sELISA)

A sELISA was developed to quantify levels of human iC3b in tissue lysates generated from the SNc of control and PD brains (see SDS-Page and Western Blotting for a description of lysate preparation). Levels of iC3b were measured using a monoclonal antibody raised against a neo-epitope in human iC3b (Quidel A209) as a capture antibody and a polyclonal anti-human C3 antisera (Quidel A304) as a detector antibody (Supplemental Fig. 2 online resource). The iC3b capture antibody was diluted to 2 ng/μl in borate saline (100 mM boric acid, 25 mM sodium tetraborate decahydrate, 75 mM NaCl, 250 μM thimerosal) and 50 μl was added to wells of high binding microtiter plates (Corning 3590) and incubated overnight at 4 °C. Wells were rinsed twice with 200 μl of ELISA wash buffer (100 mM boric acid, 25 mM sodium tetraborate decahydrate, 75 mM NaCl, 250 μM thimerosal, 0.4% bovine serum albumin, and 0.1% tween-20) and blocked with 200 μl ELISA wash buffer containing 5% nonfat dried milk (Sanalac) for 1 h at RT. Wells were then washed twice with 200 μl ELISA wash buffer. A standard curve of purified human iC3b protein (Complement Tech, A115) was generated by performing 1:3 dilutions of iC3b in TBS, with the standard curve ranging from 200 nM to 0.001 nM, and 50 μl/well were added to plates in triplicate. Human brain lysate was diluted to a final concentration of 1 μg/μl, and 50 μl/well was added to plates in duplicate. Wells containing TBS and RIPA lysis buffer blanks were included to control for non-specific signal generated from buffers. Standards, samples and blanks were incubated in wells for 2 h at RT. Wells were washed four times with 200 μl of ELISA wash buffer, after which the anti-human C3 detector antibody was diluted 1:500 in ELISA wash buffer containing 5% non-fat dry milk, and 50 μl was added to each well and incubated for 2 h at RT. Wells were washed four times with 200 μl ELISA wash buffer and then incubated with 50 μl/well of a donkey-anti goat secondary antibody conjugated to horseradish peroxidase (Jackson ImmunoResearch 705–035-003; RRID: AB_2340390), diluted 1:5,000 in ELISA wash buffer containing 5% non-fat dry milk. Wells were washed 4 times with 200 μl ELISA wash buffer and bound antibody was detected with 50 μl of 3,3’,5,5’-tetramethylbenzadine (TMB; Sigma Aldrich T0440) substrate for 2–15 min. The reaction was stopped with 50μl of 3.6% H_2_SO4 and absorbance at 450 nm (A450) was quantified with a Spectramax plus plate reader (Molecular Devices 18,780). The average A450 of blank wells was subtracted from all wells to account for non-specific background signal, and the average from standard triplicates and sample duplicates was calculated. Absorbance (A) is not linear [i.e., A = Log10(1/transmittance)], thus, the absorbance data were converted to percent absorbed light (a linear scale) using the following equation percent light absorbed = (1 − 10 − x) ∗ 100, where x is absorbance. The standard curve was Log_10_ transformed and best fit to a sigmoidal curve, thereby providing a standard curve of known amounts of iC3b protein. Samples were plotted onto the standard curve to ensure all sample values were within the linear range of the assay (Supplemental Fig. 2 online resource), and the quantity of iC3b protein (ng) in each human sample was then interpolated from the iC3b standard curve and converted to a concentration expressed in ng/μl. Graphs and concentrations were produced using GraphPad Prism 11 software.

### Immunohistochemistry

Immunohistochemistry was performed on both free-floating rat tissue and paraffin-embedded human tissue. For paraffin-embedded tissue, tissue was de-paraffinized by performing 2 × 5 min incubations in xylenes, 2 × 3 min incubations in 100% ethanol, 2 × 1 min incubations in 95% ethanol, a 1 × 1 min incubation in 70% ethanol, a 1 × 1 min incubation in 50% ethanol followed by a ~ 15 s dip in ddH2O. Tissue was then incubated in 1 × IHC Select Citrate Buffer (Millipore 21,545) at 95 °C for 10 min to expose epitopes, and cooled to RT.

All tissue (both free floating and paraffin embedded) was then washed 6 × 10 min in TBS containing 0.5% triton X-100 (TBS-Tx). Endogenous peroxidase activity was quenched by incubating tissue in 3% hydrogen peroxide diluted in TBS-Tx for 1 h, followed by 6 × 10 min washes in TBS-Tx. Tissue was blocked for 1 h in TBS-Tx containing 10% goat serum and 2% bovine serum albumin (BSA). Tissue was then incubated overnight at RT in primary antibody diluted in TBS-Tx containing 2% goat serum. Primary antibodies and concentrations used are listed in Supplemental Table 3 online resource. Following primary antibody incubation, tissue was washed 6 × 10 min in TBS-Tx. After which, the tissue was incubated in goat anti-rabbit biotinylated (Vector Laboratories, BA-1000; RRID: AB_2313606) or goat anti-mouse biotinylated (Jackson Immunoresearch115-065166; RRID AB_2338569) secondary antibodies diluted at 1:500 in TBS-Tx containing 2% goat serum for 2 h at RT. Tissue was then washed 6 × 10 min washes in TBS-Tx and in incubated in ABC Elite solution (Vector Labs, PK6100) according to manufacturer’s instructions for 2 h at RT. Tissue was washed 6 × 10 min in TBS-Tx, and bound antibodies were visualized using 3, 3’-diaminobenzidine (Sigma, D5637) at 0.5 mg/ml in TBS-Tx containing 0.003% hydrogen peroxide for 5–10 min. Tissue was then washed in TBS, mounted on microscope slides and processed through ethanol and xylenes before cover slipping with Cytoseal-60 (Thermo, 831,016). Images were acquired with a Nikon Eclipse 90i microscope, a Nikon DS-Ri1 camera and Nikon Elements Software (Nikon Instruments, Melville, NY). All individual images between animals within a particular stain were acquired using identical microscope and post-processing parameters (magnification, light source intensity, exposure time and contrast). Images were prepared for publication using Adobe Photoshop (version 26.8.1) and Illustrator (version 29.6.1).

### Immunofluorescence and quantification

Immunofluorescence staining was performed using established protocols [3, 75]. Fixed coronal sections were washed 6 × 10 min in TBS containing 0.5% Triton- × 100 (TBS-Tx). For C3 staining in rat tissue only (using Abcam ab200999), tissue was then incubated in 1 × IHC Select Citrate Buffer (Millipore 21,545) at 95 °C for 10 min to expose epitopes, cooled to RT, then washed 6 × 10 min in TBS-Tx. All tissue was blocked for 1 h at RT in TBS-Tx containing 10% goat serum and 2% bovine serum albumin. Tissue was then incubated overnight at RT in primary antibody diluted in TBS-Tx containing 2% goat serum. Primary antibodies and the concentrations used are listed in Supplemental Table 3 online resource. Following primary antibody incubation tissue was washed 6 × 10 min in TBS-Tx, and then incubated for 2 h at RT in secondary antibody diluted 1:500 in TBS-Tx containing 2% goat serum. Secondary antibodies used were goat anti-rabbit IgG Alexa Fluor 488 (Invitrogen, A11034; RRID: AB_2576217), goat anti-rabbit IgG Alexa Fluor 594 (Invitrogen, A11012; RRID: AB_2534079), goat anti-rabbit Alexa Fluor 647 (Invitrogen, A21245; RRID: AB_2535813), goat anti-mouse IgG2a Alexa Fluor 594 (Invitrogen, A21135; RRID: AB_2535774), goat anti-mouse IgG2a Alexa Fluor 488 (Invitrogen, A21131; RRID_AB_141618), Goat anti mouse IgG1 Alexa Fluor 488 (Invitrogen, A11029; RRID: AB_2534088), Goat anti-mouse IgG2b Alexa Fluor 488 (Invitrogen, A21141; RRID: AB_2535778), goat anti-mouse IgG2b Alexa Fluor 647 (Invitrogen, A21242; RRID: AB_2535811). Tissue was washed 6 × 10 min in TBS-Tx with the first of 6 washes containing 4',6-Diamidino-2-Phenylindole, Dihydrochloride (DAPI) (1:10,000, Invitrogen, D1306: RRID: 2,629,482). Sections were mounted on HistoBond + slides (VWR VistaVision, 16,004–406) and cover-slipped with VECTASHIELD hardset antifade mounting medium (Vector Laboratories, H-1400–10). Microscope slides were then imaged on a Nikon Eclipse 90i fluorescence microscope or a Nikon A + laser scanning confocal microscope and Nikon Elements Software (Nikon Instruments, Melville, NY). All individual images between animals within a particular stain were acquired using identical microscope and post-processing parameters (magnification, light source intensity, laser intensity, exposure time and contrast).

Quantification of immunofluorescent intensity was performed using FIJI (version 2.14.0/1.54f). Briefly, 10 × stitched multi-panel images containing the entire brain region of interest were imported into FIJI and a threshold was applied to exclude background fluorescence. For all immunofluorescent analyses, 3 serial sections per region of interest were analyzed for each animal. Identical thresholding parameters were applied to all images between animals within a stain. A region of interest was drawn around the anatomical boundaries of the brain regions being analyzed and the “analyze particles” function was used to quantify the fluorescent intensity and percent area of positive staining within the region of interest. Integrated density is reported as an index of fluorescent intensity. Fluorescence intensity and percent area of positive staining were averaged across the 3 sections from each animal and reported as the fold change from PBS controls. Images were prepared for publication using Photoshop (version 26.8.1) and Illustrator (version 29.6.1).

### C1q binding assay

A plate based-C1q binding assay was developed to quantify the ability of human C1q to bind human α-syn monomers and human α-syn PFFs. Human α-syn monomers were purified and fibrillized as described above. Purified human α-syn monomer, human α-syn PFF, human serum albumin (HSA; Sigma Aldrich A9511) or human IgG (Jackson ImmunoResearch 009-000-003; RRID:AB_2337043) proteins were diluted to a concentration of 12 μM in phosphate buffered saline (PBS; 137 mM NaCl, 2.7 mM KCl, 10 mM Na_2_hPO_4_, 1.8 mM KH_2_PO_4_) and serially diluted 1:3 from 12 μM to 0.2 nM in PBS. 50 μl of the respective dilutions were added to wells of high binding microplates (Corning 3590) and incubated overnight at 4 °C with gentle shaking. Wells were washed 3 times with 200 μl PBS containing 0.05% tween-20 (PBS-T), then blocked with 200 μl of 5% non-fat dry milk (Sanalac) diluted in PBS for 1 h at RT with gentle shaking. Wells were washed 3 times with 200 μl PBS-T. Purified human C1q (complement technology A099) was diluted to 2 μg/mL in GVB++ buffer (complement technology B102) pre-heated to 37 °C and 50 μl was added to wells and incubated for 1 h at 37 °C with gentle shaking. Wells were washed 4 times with 200 μl PBS-T and then incubated in 50 μl of a monoclonal anti-human C1q primary antibody (1:2,000, Invitrogen MA1-40,311; RRID:AB_2067275) diluted in 5% non-fat dry milk for 1 h at RT with gentle shaking. Wells were washed 4 × with 200 μl PBS-T and then incubated in 50 μl of goat anti-mouse horse radish peroxidase conjugated secondary antibody (1:5,000; Jackson ImmunoResearch 115-035-003; RRID:AB_100015289) diluted in 5% non-fat dry milk for 1 h at RT with gentle shaking. Wells were washed 4 × with 200 μl PBS-T and then bound antibodies were visualized with 50 μl of 3,3’,5,5’-tetramethylbenzadine (TMB; Sigma Aldrich T0440) substrate for 5–15 min. The reaction was stopped with 50 μl of 3.6% H_2_SO4 and absorbance at 450 nm (A450) was quantified with a Spectramax plus plate reader (Molecular Devices 18,780). The average A450 of blank wells (containing only 5% non-fat dry milk) was subtracted from all wells to account for non-specific background signal. As above, the absorbance data were converted to percent absorbed light (a linear scale) using the following equation percent light absorbed = (1 – 10 − x) ∗ 100, where x is absorbance. Graphs and EC50 concentrations were produced using GraphPad Prism software.

### Complement activation assay

A plate-based complement activation assay was developed to quantify the ability of human α-syn monomers or human α-syn PFFs to activate the complement system. Human α-syn monomers were purified and fibrillized as described above. Purified human α-syn monomer, human α-syn PFF, human serum albumin (HSA: Sigma Aldrich A9511) or human IgG (Jackson Immunoresearch 009-000-003; RRID:AB_2337043) proteins were diluted to a concentration of 4 μM in phosphate buffered saline (PBS; 137 mM NaCl, 2.7 mM KCl, 10 mM Na_2_hPO_4_, 1.8 mM KH_2_PO_4_) and serially diluted 1:3 from 4 μM to 0.067 nM in PBS. Samples (50 μl/well) were added to high binding 96 well microplates (Corning 3590) and incubated overnight at 4 °C with gentle shaking. The following day, wells were washed 3 times with 200 μl PBS containing 0.05% tween-20 (PBS-T) and then blocked in 2% bovine serum albumin (BSA; Fisher BP1600-100) diluted in PBS for 1 h at RT. Wells were washed three times with 200 μl PBS-T and then incubated in 2% normal human serum (NHS; Complement Technology, NHS) diluted in GVB++ buffer (complement technology B102) at 37 °C for 1 h with gentle shaking. Wells were then washed four times with 200 μl of PBS-T and incubated in 50 μl of a primary antibody specific to a neo-epitope in cleaved (activated) complement C3 (1:500; Hycult HM2257; RRID:AB_1953566) diluted in 2% BSA for 1 h at RT with gentle shaking. Wells were washed 4 × with 200 μl PBS-T and then incubated in 50 μl of goat anti-mouse horse radish peroxidase conjugated secondary antibody (1:5,000, Jackson Immunoresearch 115-035-003; RRID:AB_100015289) diluted in 2% BSA for 1 h at RT with gentle shaking. Wells were washed 4 × with 200 μl PBS-T and bound antibodies detected with 50 μl of 3,3’,5,5’-tetramethylbenzadine (TMB; Sigma Aldrich T0440) substrate for 5–15 min. The reaction was stopped with 50 μl of 3.6% H_2_SO_4_ and absorbance at 450 nm (A450) was quantified with a Spectramax plus plate reader (Molecular Devices 18,780). As above, absorbance data were converted to percent absorbed light (a linear scale) using the following equation percent light absorbed = (1 − 10 − x) ∗ 100, where x is absorbance. Graphs and EC50 concentrations were produced using GraphPad Prism 11 software.

### Statistical analysis

Statistical analysis and graphing of results were performed using GraphPad Prism 11. For ddPCR and biochemical endpoints in rat tissue, data from both sexes were compiled, and a two-way ANOVA with a Tukey’s multiple comparison test was used to compare differences between sexes and treatment. If there was no significant difference between sexes within a treatment group, sexes were combined and data was analyzed with either an unpaired t-test and Welch’s correction to determine differences between two groups (i.e. PBS, PFF) or a one-way ANOVA with Tukey’s multiple comparison test to determine differences between three groups (i.e. PBS, PFF, monomer). Statistical data for all analyses that were used to determine sex differences is included in the Supplemental Tables 4, 5 online resources. Immunofluorescence analysis of C3, CR3 and NPTX1 was performed to localize the cell source and measure protein of targets found to be significantly altered by ddPCR and immunoblotting. As no significant sex differences were detected in these targets by ddPCR or immunoblotting (Supplemental Tables 4, 5 online resources), immunofluorescent analysis was performed on mixed sexes. For concentration response analyses in the in vitro C1q binding assay and complement activation assay, data were analyzed using a two-way ANOVA with Tukey’s multiple comparison. Statistical analysis of EC50 and maximum binding/maximum activation calculated for C1q binding and complement activation assays, was performed with a one-way ANOVA and Tukey’s multiple comparison test. In all cases significance was set at *p* ≤ 0.05.

## Results

**Fig. 2:**
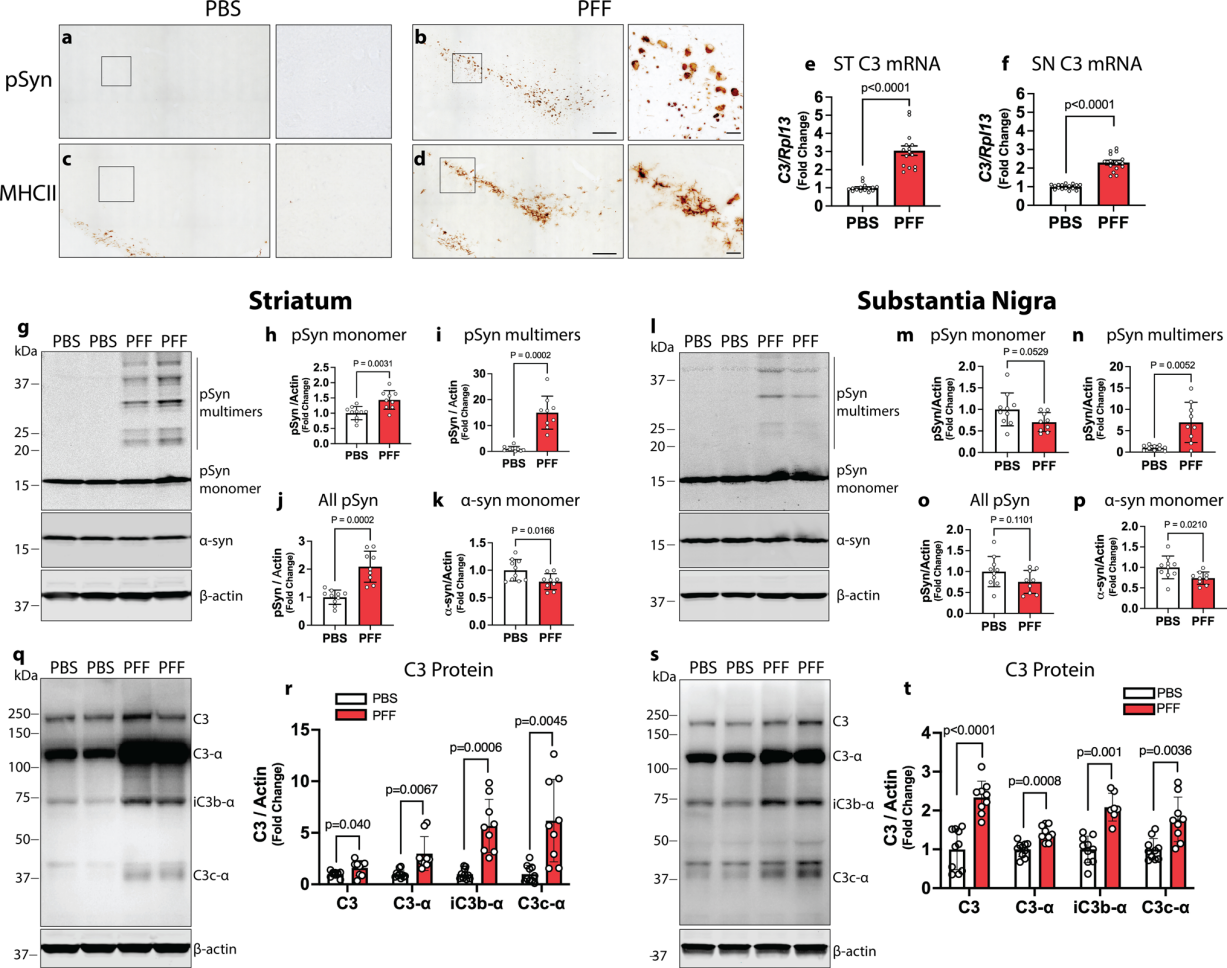
Increased C3 expression and activation in the nigrostriatal tract of α-syn PFF injected rats. Rats (*n* = 6–8/sex/group for ddPCR and *n* = 4–5/sex/group for biochemistry) received intra-striatal injections of mouse α-synuclein (α-syn) preformed fibrils (PFFs) or phosphate buffer saline (PBS) and sacrificed 2-months post-injection. No significant sex differences were detected within any endpoints (Supplementary Tables 4, 5), thus, sexes were combined within groups for analyses. **a**, **b** Serine 129 phosphorylated α-syn (pSyn) immunostaining in the substantia nigra (*SN*) of PBS (**a**) and α-syn PFF (**b**) injected rats. **c**, **d** MHC-II immunostaining in the SN of PBS (**c**) and α-syn PFF (**d**) injected rats. High magnification panels correspond to the area in box of low magnification images to the left. **e**, **f** Quantification of *C3* expression (normalized to *Rpl13*) in the striatum (*ST*; **e**) and SN (**f**), analyzed with *t*-test with Welch’s correction. **g**, **l** Immunoblot of pSyn, α-syn and β-actin from ipsilateral ST (**g**) and SN (**l**). **h**–**k** Quantification of pSyn monomers (~ 14–17 kDa; **h**), pSyn multimers (~ 20–50 kDa; **i**), total pSyn (~ 14–50 kDa; **j**), and α-syn monomer (~ 14–17 kDa; **k**) normalized to β-actin in the ST. **m**–**p** Quantification of pSyn monomers (~ 14–17 kDa; **m**), pSyn multimers (~ 20–50 kDa; **n**), total pSyn (~ 14–50 kDa; **o**), and α-syn monomer (~ 14–17 kDa; **p**) normalized to β-actin in the SN. **q**, **s** Immunoblot of C3 and β-actin from the ipsilateral ST (**q**) and SN (**s**). Quantification of intact C3 (~ 190 kDa), C3 α-chain (~ 115 kDa), iC3b α-chain (~ 75 kDa), and C3c α-chain (~ 34 kDa) normalized to β-actin from the ST (**r**) and SN (**t**). C3, pSyn and α-syn analyzed with t-test with Welch’s correction. Scale bars in large images of (**b**, **d**) are 250 μm and apply to large images of (**a**–**d**). Scale bar in small images of (**b**, **d**) are 50 μm and apply to small images of (**a**–**d**). All data are group means ± standard deviation expressed a fold change from PBS group

We aimed to determine if pathological α-syn activates the complement system prior to neurodegeneration using a rat model of synucleinopathy. Two months post-α-syn PFF injection represent the time of peak pathological α-syn accumulation and peak glial activation, but months prior to overt degeneration of nigrostriatal dopamine neurons [12, 56, 75, 76]. A subset of animals was used to confirm successful seeding of endogenous α-syn into phospho-serine 129 α-syn (pSyn) + aggregates and microglial activation (as indicated by MHC-II expression) in the ipsilateral SNc. In line with previous reports [12, 56], the ipsilateral SNc of α-syn PFF injected rats contained abundant pSyn + aggregates (Fig. 2b), and MHC-II + microglia (Fig. 2d), while no pSyn + aggregates or MHC-II + microglia were observed in the SNc of PBS injected rats (Fig. 2a, c). Next, we quantified *C3* gene expression in the SN and ST and detected a significant increase in *C3* expression in both brain regions of PFF injected rats compared to PBS controls (Fig. 2e, f), validating and extending previous findings [75].

To determine if pathological α-syn activates the complement system prior to degeneration we performed biochemical quantification of pSyn (as a surrogate marker of pathological α-syn) and C3 in the SN and ST, but we first confirmed the specificity of the antibody used to quantify pSyn (Supplemental Fig. 3 online resource). There was a significant increase in the amount of pSyn monomer (~ 14–17 kDa band) and pSyn multimers (~ 20–50 kDa bands) in the ST of PFF injected rats (Fig. 2g, h, i), while levels of α-syn monomer were significantly decreased in the ST of PFF injected rats (Fig. 2g, k). In the SN, pSyn multimers were significantly increased (Fig. 2l, n), while α-syn monomers were significantly reduced (Fig. 2l, p).

Complement C3 sits at a functional nexus in the complement system (Fig. 1b) and complement proteins are activated by proteolytic cleavage. Thus, to quantify complement activation in the ST and SN of PBS and PFF injected rats, we performed immunoblotting to quantify intact C3 (as indexed by whole molecule C3 (~ 190 kDa), the α-chain of intact C3 (~ 115 kDa), the α-chain of iC3b (~ 68 kDa; an activated complement opsonin derived from C3) and the α-chain of C3c (~ 35–40 kDa; a downstream product of C3 activation). In line with C3 gene expression (Fig. 2e, f), overall levels of C3 protein were significantly increased in the ST (Fig. 2q, r) and SN (Fig. 2s, t) of α-syn PFF injected rats. Further, levels of iC3b and C3c were significantly increased in the ST and SN of PFF injected rats compared to PBS controls, confirming complement activation in response to α-syn PFF injection (Fig. 2q–t). Finally, we quantified C3 and its activation products in the SN and ST of rats injected with α-syn monomer control protein to determine if C3 activation in α-syn PFF injected rats reflects a response to synucleinopathy or the injection of exogenous α-syn protein (Supplemental Fig. 4 online resource). In the SN, intact C3 levels were significantly increased in α-syn monomer injected rats compared to PBS controls, but were significantly lower than in PFF injected rats. There was no change in levels of C3 activation products in the SN of α-syn monomer injected rats compared to PBS controls. In the ST, C3c levels were significantly increased in α-syn monomer injected rats compared to PBS controls, but again were significantly lower than in PFF-injected rats. No other significant changes in C3 or its activation products were detected in α-syn monomer injected rats. Importantly, levels of C3 and C3 activation products were significantly elevated in α-syn PFF injected rats compared to α-syn monomer injected rats, indicating that complement activation in α-syn PFF injected rats is primarily driven by aggregates of endogenous α-syn rather than injection of exogenous protein alone. Together, these data show that injection of α-syn PFFs in the ST of rats induces the formation of pSyn + multimers in the ST and SN that corresponds with a shift of soluble α-syn monomers to insoluble α-syn aggregates, and an associated increase in C3 activation in the ST and SN.

**Fig. 3:**
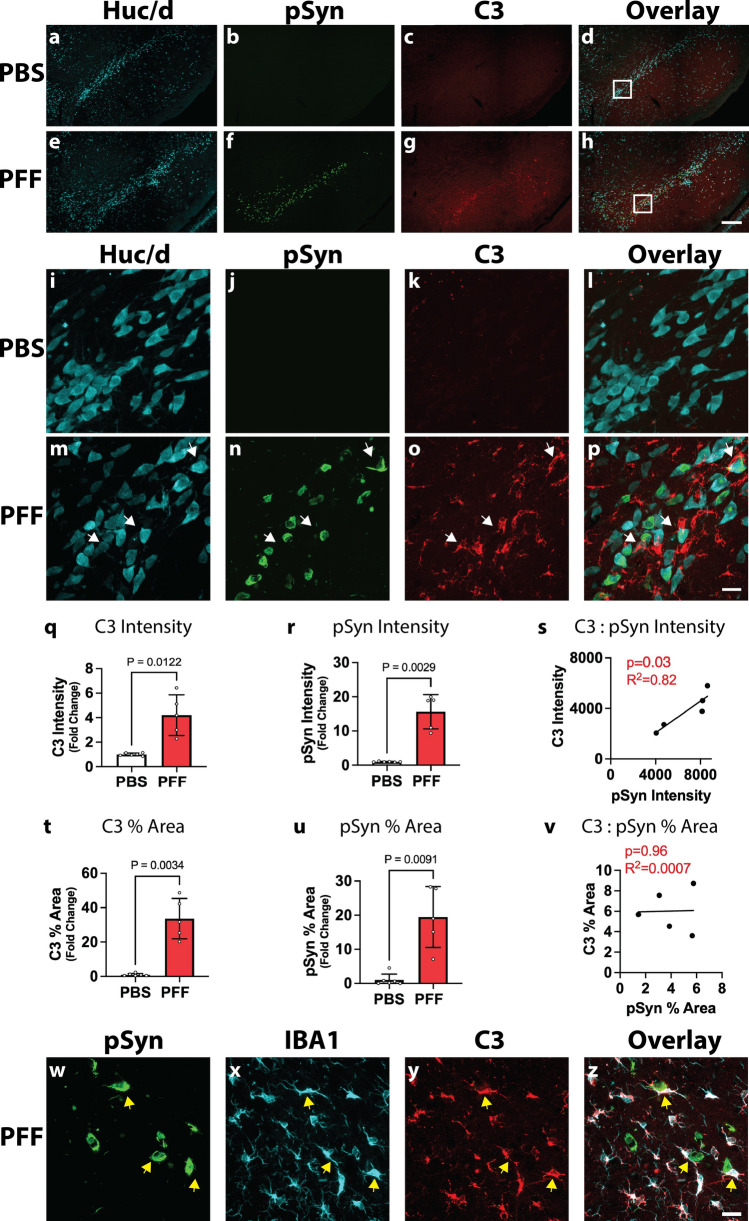
Microglia upregulate C3 in the substantia nigra of α-syn PFF injected rats. Male and female rats (*n* = 5–6/group) received intra-striatal injections of mouse α-synuclein (α-syn) preformed fibrils (PFFs) or phosphate buffer saline (PBS) and were sacrificed 2 months post injection. No significant sex differences were detected in levels of C3 by ddPCR or immunoblotting (Supplementary Tables 4, 5), thus immunofluorescent (IF) analysis was performed on mixed sexes. **a**–**h** Representative low magnification IF images of Huc/d (neuronal marker, cyan), serine 129 phosphorylated α-syn (pSyn; green) and complement component 3 (*C3*; red) and the overlay image in the SNc of PBS (**a**–**d**) and PFF (**e**–**h**) injected rats. **i**–**p** High magnification images corresponding to box in (**d**) and (**h**), for PBS (**i**–**l**) and PFF injected (**m**–**p**) rats, respectively. **q**, **r** Quantification of C3 (**q**) and pSyn (**r**) fluorescence intensity. **s** Regression analysis between pSyn and C3 fluorescence intensity in the SNc of PFF injected rats. **t**, **u** Quantification of percent area of SNc occupied by C3 + (**t**) and pSyn + (**u**) staining. **v** Regression analysis between pSyn and C3 percent area staining in the SNc of PFF injected rats. Data expressed as mean fold change (± standard deviation) from PBS controls, analyzed by t-test with Welch’s correction or simple linear regression analyses. **w**–**z** Representative IF images of pSyn (green; **w**), the microglial marker, ionized calcium binding adapter molecule 1 (IBA1; cyan; **x**), C3 (red; **y**) and the overlay image (**z**) in the SNc of PFF injected rats. Arrows in (**m**–**p**) and (**w**–**z**) indicate areas where C3 + microglia are in direct apposition of neurons containing pSyn + aggregates. Scale bars in (**h**) is 250μm and applies to (**a**–**h**), scale bar in (**p**) is 50μm and applies to (**i**–**p**), scale bar in (**z**) is 50μm and applies to (**w**–**z**)

We next sought to identify the cell source upregulating C3 in α-syn PFF injected rats using immunofluorescent (IF) detection of C3, pSyn and the neuronal marker, Huc/d. No pSyn immunoreactivity and very few C3 + cells were observed in the ventral midbrain of PBS injected rats (Fig. 3a–d, i–l), where the majority of C3 + cells were located outside of the SNc, primarily in the substantia nigra pars reticulata (SNr) and cerebral peduncles of the ventral midbrain (Fig. 3c). In contrast, abundant pSyn + inclusions were present throughout the SNc of α-syn PFF injected rats, which was associated with a dramatic increase in the number of C3 + cells in the SNc (Fig. 3e–h, m–p). The upregulation of C3 in the midbrain of PFF injected rats spatially overlapped with pSyn aggregates in the SNc, but increased C3 + cells were also observed in the SNr and areas adjacent to the SNc. High magnification confocal imaging of the pSyn seeded SNc shows abundant C3 + cells in direct apposition to neurons containing pSyn aggregates (arrows in Fig. 3m–p), suggesting C3 + cells are targeting synucleinopathy-affected neurons. C3 fluorescence intensity and the percent area of C3 staining were significantly increased in the SNc of α-syn PFF injected rats (Fig. 3q, t), and this was paralleled by significant increases in pSyn IF intensity and percent area staining (Fig. 3r, u). In α-syn PFF injected rats, C3 IF intensity was significantly correlated with pSyn IF intensity (Fig. 3s). Finally, we identified microglia as the primary cellular source of C3 in the SNc of PFF injected rats, as indicated by high colocalization between C3 with IBA1 (Fig. 3w–z) and lack of C3 colocalization with GFAP, in the brains of PFF injected rats (Supplemental Fig. 5 online resource). These data are in line with the biochemical quantification of C3 and pSyn in PFF injected rats (Fig. 2) and, utilizing complementary endpoints, validate the association between neuronal pSyn pathology and microglial C3 upregulation during the early phases of synucleinopathy in α-syn PFF injected rats.

**Fig. 4:**
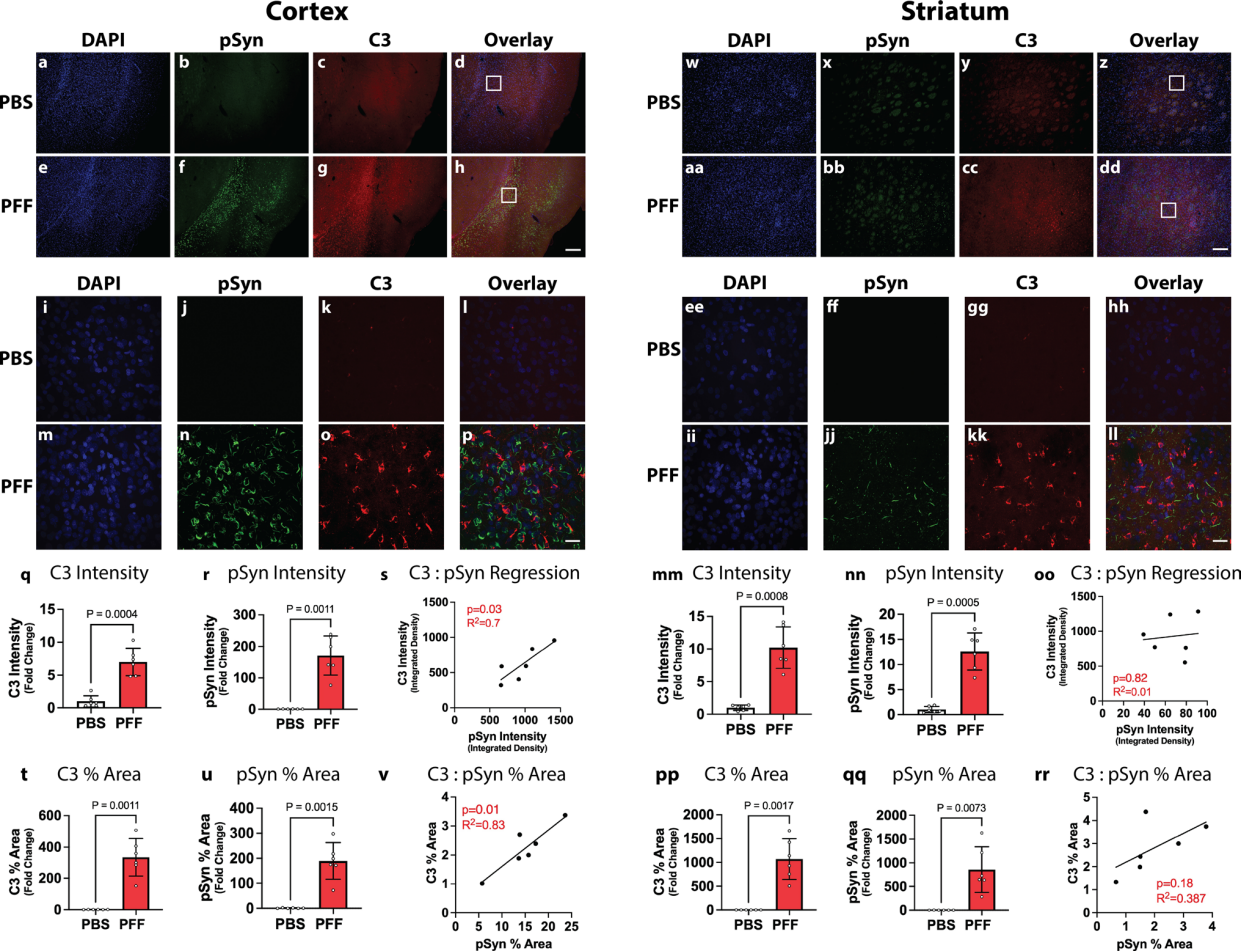
C3 upregulation in the striatum and cortex of α-syn PFF injected rats. Male and female rats (*n* = 5–6/group) received intra-striatal injections of mouse α-synuclein (α-syn) preformed fibrils (PFFs) or phosphate buffer saline (PBS) and sacrificed 2-months post-injection. All analyses were performed on groups composed of mixed sexes. **a**–**h** Representative low magnification images of DAPI (nuclear marker, blue), serine 129 phosphorylated α-syn (pSyn; green) and complement 3 (C3; red) and the overlay in the ipsilateral cortex (Cx) of PBS (**a**–**d**) and α-syn PFF (**e**–**h**) injected rats. **i**–**p** High magnification images corresponding to box in (**d**) and (**h**), for PBS (**i**–**l**) and PFF (**m**–**p**) injected rats, respectively. **q**, **r** Quantification of C3 (**q**) and pSyn (**r**) fluorescence intensity in the Cx. **s** Linear regression between pSyn and C3 fluorescence intensity in the Cx of PFF injected rats. **t**, **u** Quantification of percent area of Cx occupied by C3 + (**t**) and pSyn + (**u**) staining. **v** Linear regression between pSyn and C3 percent area staining in the Cx of PFF injected rats. **w**–**dd**) Representative low magnification images of DAPI (blue), pSyn (green) and C3 (red) and overlay image in the ipsilateral striatum (ST) of PBS (**w**–**z**) and PFF (**aa**–**dd**) injected rats. **ee**–**ll** High magnification images corresponding to box in (**z**) and (**dd**), for PBS (**ee**–**hh**) and PFF (**ii**–**ll**) injected rats, respectively. **mm**, **nn** Quantification of C3 (**mm**) and pSyn (**nn**) fluorescence intensity in the ST. **oo** Linear regression between pSyn and C3 fluorescence intensity in the ST of PFF injected rats. **pp**, **qq** Quantification of percent area of ST occupied by C3 + (**pp**) and pSyn + (**qq**) staining. **rr** Linear regression between pSyn and C3 percent area staining in the ST of PFF injected rats. Data expressed as mean fold change (± standard deviation) from PBS controls (analyzed with unpaired t-test with Welch’s correction or simple linear regression analyses). Scale bars in (**h**; **dd**) are 250 μm and apply to (**a**–**h**; **w**–**dd**), respectively. Scale bars in (**p**; **ll**) are 50 μm and apply to (**i**–**p**; **ee**–**ll**), respectively

Next, we sought to determine if microglial C3 upregulation in α-syn PFF injected rats is unique to the SNc or if it also occurs in other brain regions that accumulate pathological α-syn (i.e., the cortex (Cx) and ST) [56, 59]. In α-syn PFF injected rats, pSyn immunoreactivity was significantly increased in the Cx (Fig. 4e–h, m–p; at the level of the claustrum, endopiriform nucleus, agranular and piriform areas) and ST (Fig. 4aa–dd, ii–ll). As previously described [56], pSyn immunoreactivity in the Cx appears as concentrated aggregates in neuronal somata (Fig. 4n, p), while pSyn immunoreactivity in the ST appears as pSyn + neuronal processes resembling Lewy neurites, reflecting pathological α-syn in the terminals of nigrostriatal and corticostriatal neurons (Fig. 4jj, ll). Regardless of the brain region or morphology of pSyn aggregates, there was a significant increase in C3 + cells in both the Cx (Fig. 4g, o) and ST (Fig. 4cc, kk) of PFF injected rats. Compared to PBS injected controls, both C3 and pSyn IF intensity and percent area staining were significantly increased in the Cx (Fig. 4q, r, t, u) and ST (Fig. 4mm, nn and pp, qq) of PFF injected rats. Finally, in α-syn PFF injected rats C3 IF intensity and percent area staining significantly correlated to pSyn IF intensity and percent area staining in the Cx (Fig. 4s, v) but not the ST (Fig. 4oo, rr). These data suggest microglia upregulate C3 in response to pathological α-syn across affected brain regions.

However, while the upregulation of C3 was similar between the Cx and SNc (~ 6 fold increase from controls in Cx versus ~ 5 fold increase from controls in the SNc), there is significantly more pSyn pathology in the Cx versus the SNc (~ 120 fold increase in the Cx versus ~ 15 fold increase from controls in the SNc). Thus, there may be a disproportional upregulation of C3, relative to pSyn levels, in the SNc when compared to other regions. This is also true in the ST, however it is possible that some changes in the ST may be in response to the injectate itself (i.e. exogenous α-syn protein) as opposed to endogenous α-syn seeded into aggregates. As the SNc and Cx are physically separated from the injection site, this potential confound does not apply, suggesting an exaggerated complement response to Lewy body-like α-syn aggregates in the SNc. This differential response could potentially contribute to the selective vulnerability of nigral dopamine neurons to PD-associated pathology.

**Fig. 5:**
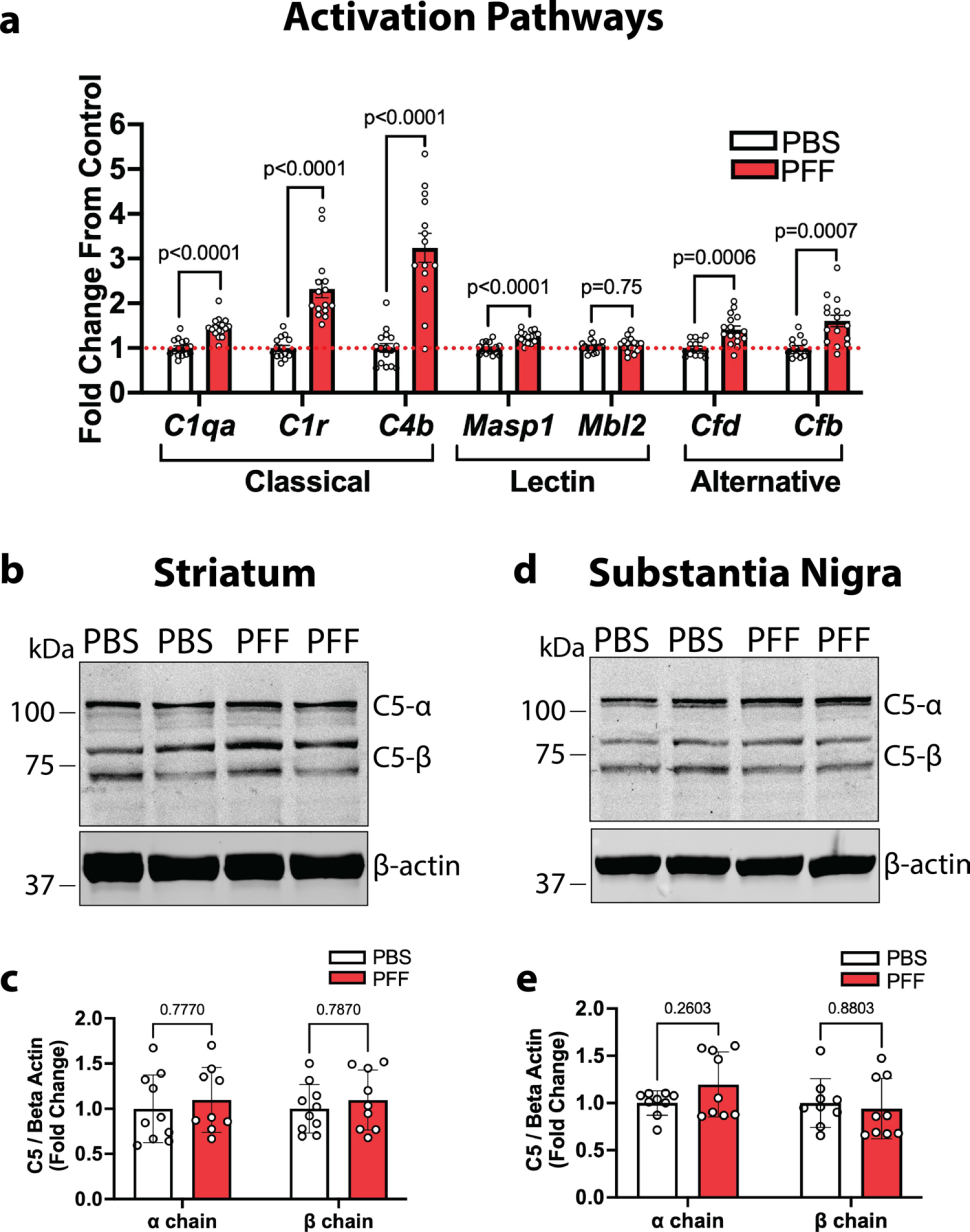
Preferential upregulation of the classical pathway in the substantia nigra of α-syn PFF injected rats. Male and female rats (*n* = 6–8/sex/group for ddPCR and *n* = 4–5/sex/group for biochemistry) received intra-striatal injections of mouse α-synuclein (α-syn) preformed fibrils (PFFs) or phosphate buffer saline (PBS) and were sacrificed 2-months post-injection. For all endpoints, no significant sex differences were detected within any group (Supplementary Tables 4, 5), thus sexes were combined within groups for all analyses. **a** Droplet digital PCR (ddPCR) quantification of transcripts representing targets of the classical (*C1qa, C1r, C4b*), lectin (mannan-associated serine protease 1 (*Masp1),* mannose binding lectin 2 *(Mbl2*)) and alternative (complement factor D (*Cfd),* complement factor B *(Cfb*)) complement activation pathways in the ipsilateral substantia nigra (*SN*) from PBS and PFF injected rats. Data normalized to ribosomal protein L13 (*Rpl13*) and expressed as mean fold change (± standard deviation) from PBS controls (analyzed with multiple unpaired *t*-tests with Welch’s correction). **b**–**e** Immunoblotting of complement C5 (C5) in the ipsilateral striatum (ST; **b**, **c**) and SN (**d**, **e**) from PBS and PFF injected rats. **b**, **d** Representative immunoblots of C5 and β-actin in the ST (**b**) and SN (**d**) of PBS and PFF injected rats. **c**, **e** Quantification of C5 α-chain (~ 115 kDa) and β-chain (~ 70, 76 kDa) normalized to β-actin in the ST (**c**) and SN (**e**). Data are expressed as a mean fold change (± standard deviation) from the PBS controls (analyzed with multiple unpaired t tests with Welch’s correction)

C3 activation can be stimulated by one of the three complement activating pathways: classical, lectin and alternative (Fig. 1a). To determine which activating pathways are upregulated during the early phases of synucleinopathy, we quantified transcripts representing key targets within the different activation pathways using ddPCR. Specifically, we quantified genes representing the classical pathway (*C1qa*, *C1r* and *C4b*), the lectin pathway (mannan binding lectin serine protease 1 (*Masp1)* and mannose binding lectin 2 (*Mbl2)*) and the alternative pathway (complement factor D (*Cfd*) and complement factor B (*Cfb*)) in the ipsilateral SN of PBS and PFF injected rats (Fig. 5a). Expression of the classical pathway transcripts were all significantly increased in the SN of PFF injected rats compared to PBS controls. The expression of the lectin pathway target, *Masp1*, was significantly increased in the SN of PFF injected rats compared to PBS controls, however the magnitude of this increase (~ 1.2-fold) was substantively lower than the magnitude of change in genes of the classical pathway (~ 1.6–3.5 fold). Finally, expression of the alternative pathway targets were significantly increased (~ 1.5 fold), albeit to a lesser extent than the classical pathway targets (Fig. 5a). These data suggest pathological α-syn upregulates genes associated with the classical pathway and, to a lesser extent, the alternative pathway.

We next determined if there is activation or upregulation of the terminal pathway during the early phases of synucleinopathy in α-syn PFF injected rats. We used ddPCR to quantify the expression of the terminal pathway genes, *C5*, *C8a* and *C9* and immunoblotting to measure C5. Transcripts encoding the terminal pathway genes, *C5*, *C8a* and *C9* were undetectable in the ST and SN of both PBS and PFF injected rats, yet these targets were detectable in the liver (Supplemental Fig. 6 online resource), confirming functionality of the ddPCR primer/probes pairs used. Despite the lack of quantifiable *C5* transcript in the brain, we detected C5 protein by immunoblot in both the ST and SN (Fig. 5b–e), but there was no difference in overall levels of C5 protein or C5 activation products between PBS and PFF injected rats in either the ST or SN. These data suggest the terminal pathway is not activated during the early phases of synucleinopathy.

**Fig. 6:**
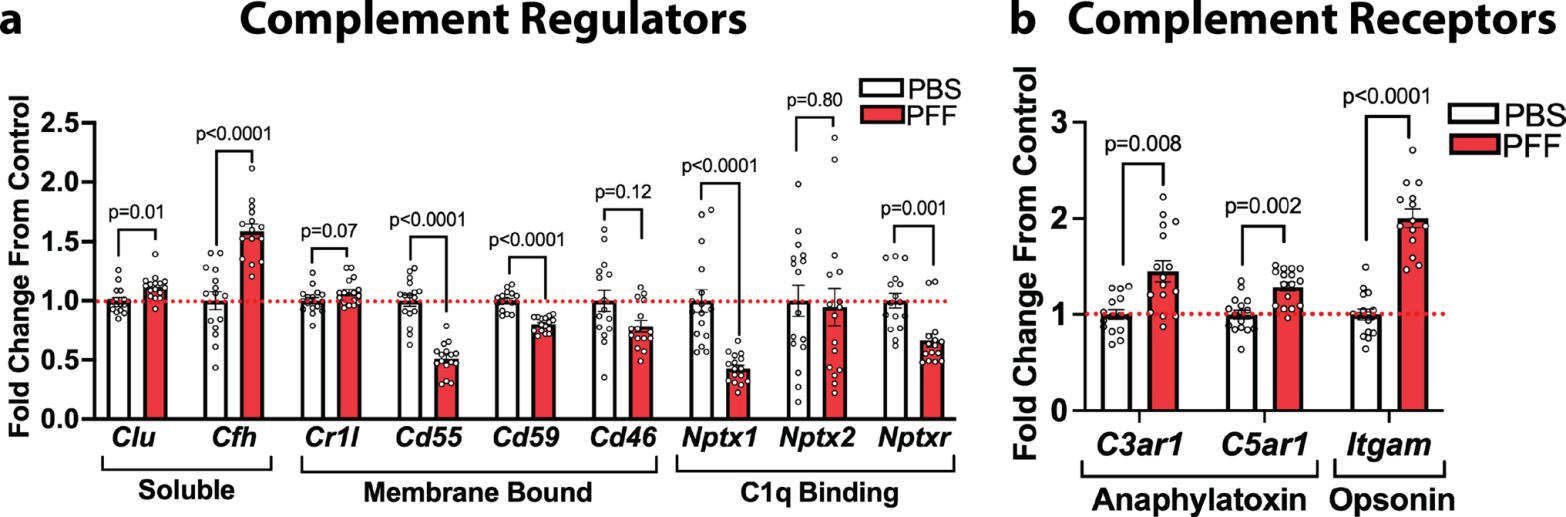
Complement receptor and regulator expression are altered in the substantia nigra of α-syn PFF injected rats. Male and female rats (*n* = 6–8/sex/group) received intra-striatal injections of mouse α-synuclein (α-syn) preformed fibrils (PFFs) or phosphate buffer saline (PBS) and were sacrificed 2-months post-injection. For all endpoints, no significant sex differences were detected within any group (Supplementary Tables 4, 5), thus sexes were combined within groups for all analyses. **a** Droplet digital PCR (ddPCR) quantification of transcripts encoding complement regulators, including the soluble regulators, clusterin (*Clu*) and complement factor H (*Cfh*); membrane bound regulators, including complement receptor 1 like protein (*Cr1l*), *Cd55*, *Cd59* and *Cd46*; C1q binding proteins, including neuronal pentraxin 1 (*Nptx1*), neuronal pentraxin 2 (*Nptx2*), and the neuronal pentraxin receptor (*Nptxr*) in the ipsilateral SN of PBS and PFF injected rats. **b** ddPCR quantification of transcripts encoding the anaphylatoxin receptors, *C3ar1 *and *C5ar1*, and the *Itgam* gene encoding a subunit of the opsonin receptor, complement receptor 3, in the ipsilateral SN of PBS and PFF injected rats. Data normalized to ribosomal protein L13 (*Rpl13*) and expressed as mean fold change (± standard deviation) from PBS controls (data analyzed with multiple unpaired t-tests with Welch’s correction)

The complement system is regulated at multiple levels by complement regulatory proteins while specific receptors mediate many of the complement effector responses. We used ddPCR to quantify the expression of select genes representing the major complement regulators and complement receptors in the SN of PBS or α-syn PFF-injected rats (Fig. 6). The specific complement regulatory genes quantified include the soluble complement regulators (clusterin (*Clu*) and complement factor H (*Cfh*)), the membrane bound complement regulators (complement receptor 1 like protein (*Cr1l*), *Cd55*, *Cd59* and *Cd46*), and the C1q binding proteins (neuronal pentraxin 1 (*Nptx1*), *Nptx2* and the Nptx-receptor (*Nptxr*)) [93]. Expression of the soluble complement regulators, *Clu* and *Cfh*, were both significantly increased in the SN of PFF injected rats compared to PBS injected rats (Fig. 6a). In contrast, expression of the membrane bound complement regulators, *Cd55* and *Cd59*, and the C1q binding proteins, *Nptx1* and *Nptxr*, were significantly decreased in the SN of PFF injected rats (Fig. 6a). Next, we quantified the expression of genes encoding the anaphylatoxin receptors, *C3ar1* and *C5ar1*, as well as the *Itgam* gene, encoding the integrin alpha M subunit of the opsonin receptor, complement receptor 3 (CR3). *C3aR1*, *C5aR1* and *Itgam* expression were all significantly increased in the SN of PFF injected rats compared to PBS injected controls (Fig. 6b). These data implicate the complement effector responses of opsonization (mediated through CR3) and inflammation (mediated through C3aR1 and C5aR1) and dysregulation of the complement system in response to synucleinopathy.

**Fig. 7:**
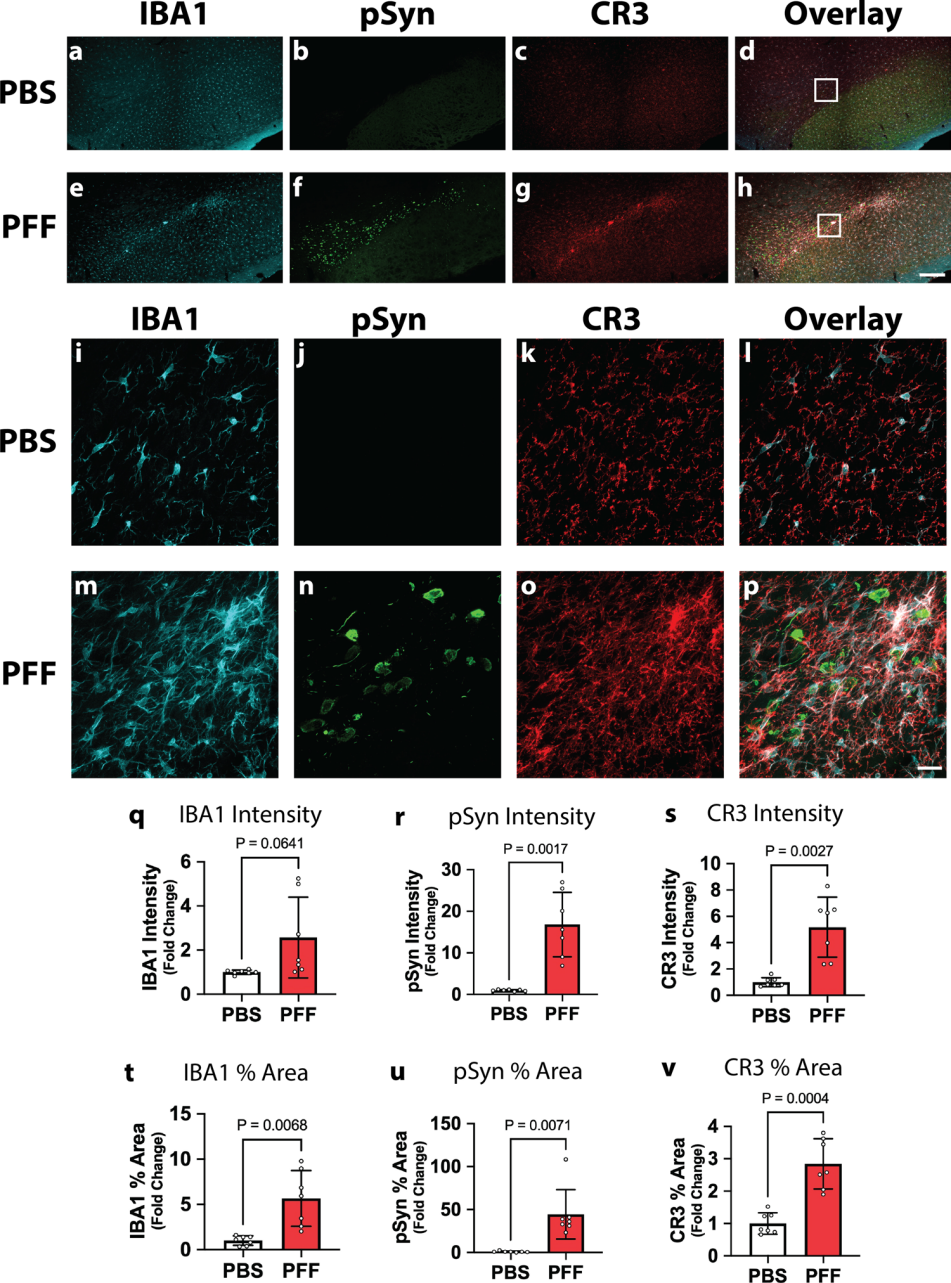
Microglial complement receptor 3 expression is increased in the substantia nigra of α-syn PFF injected rats. Male and female rats (*n* = 6–7/group) received intra-striatal injections of mouse α-synuclein (α-syn) preformed fibrils (PFFs) or phosphate buffer saline (PBS) and were sacrificed 2-months post-injection. No significant sex differences were detected in levels of CR3 (*Itgam* subunit) by ddPCR (Supplementary Table 4), thus immunofluorescent (*IF*) analysis was performed on mixed sexes. **a**–**h** Representative low magnification IF images of the microglial marker, ionized calcium binding adaptor molecule 1 (IBA1; cyan), serine 129 phosphorylated α-syn (*pSyn*; green), complement receptor 3 (*CR3*; red) and the corresponding overlay image in the SNc of PBS (**a**–**d**) and PFF (**e**–**h**) injected rats. **i**–**p** High magnification images corresponding to boxes in (**d**) and (**h**), for PBS (**i**–**l**) and PFF injected (**m**–**p**) rats, respectively. **q**–**s** Quantification of IBA1 (**q**), pSyn (**r**) and CR3 (**s**) fluorescence intensity in the SNc. **t**–**v** Quantification of the percent area of the SNc occupied by IBA1 + (**t**), pSyn + (**u**) and CR3 + (**v**) staining. Data expressed as mean fold change (± standard deviation) from PBS controls (data analyzed with *t*-test with Welch’s correction). Scale bar in (**h**) is 250μm and applies to (**a**–**h**), scale bar in (**p**) is 50μm and applies to (**i**–**p**)

We next sought to assess changes in a representative complement receptor, for which we selected CR3, at the protein level using IF in midbrain tissue from PBS and α-syn PFF-injected rats (Fig. 7). CR3 and IBA1 immunoreactivity were present throughout the ventral midbrain of PBS injected rats (Fig. 7a–d, i–l), however CR3 and IBA1 signal were dramatically increased in PFF injected rats, specifically in areas of the SNc containing pSyn aggregates, where CR3 + microglia were observed in direct apposition to pSyn + aggregates (Fig. 7e–h, m–p), suggesting CR3 + microglial are targeting neurons containing pathological α-syn. Quantification of IBA1 (Fig. 7q) and CR3 (Fig. 7s) IF intensity and the percent area of the SNc occupied by IBA1 + (Fig. 7t) and CR3 + (Fig. 7v) staining were both significantly increased in the SNc of PFF injected rats, and this increase paralleled increased pSyn levels (Fig. 7r, u). These data indicate microglia upregulate expression of the opsonin receptor, CR3, in response to pathological α-syn.

**Fig. 8:**
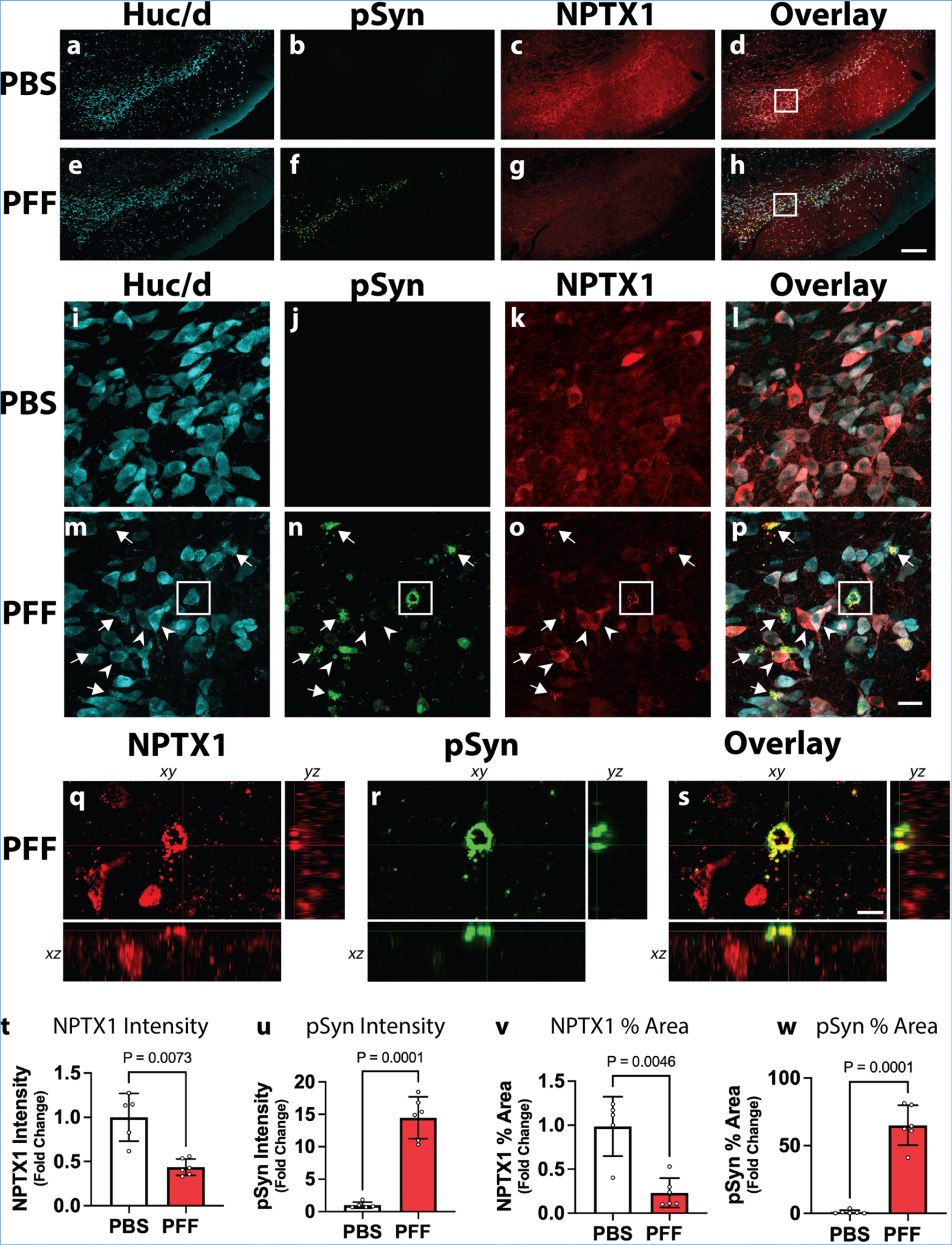
Neuronal pentraxin 1 is decreased and localized to pSyn + inclusions in nigral neurons of α-syn PFF injected rats. Male and female rats (*n* = 5–6/group) received intra-striatal injections of mouse α-synuclein (α-syn) preformed fibrils (PFFs) or phosphate buffer saline (PBS) and were sacrificed 2-months post-injection. No significant sex differences were detected in levels of *Nptx1* by ddPCR (supplementary Table 4), thus immunofluorescent (*IF*) analysis was performed on mixed sexes. **a**–**h** Representative low magnification IF images of the pan-neuronal marker, Huc/d (cyan), serine 129 phosphorylated α-syn (*pSyn*; green), neuronal pentraxin 1 (*NPTX1*; red) and the corresponding overlay image in the SNc of PBS (**a**–**d**) and PFF (**e**–**h**) injected rats. **i**–**p** Representative high magnification images corresponding to boxes in (**d**) and (**h**)**,** for PBS (**i**–**l**) and PFF injected (**m**–**p**) rats, respectively. Arrows in (**m**–**p**) indicate areas where NPTX1 co-localizes with pSyn + aggregates. Arrowheads in (**m**–**p**) indicate neurons with diffuse NPTX1 immunoreactivity and minimal or absent pSyn + inclusions. **q**–**s** Representative orthogonal view Z-stacks corresponding to the box in (**m**–**p**) showing colocalization between NPTX1 and pSyn in an aggregate. **t**–**u** Quantification of NPTX1 (**t**) and pSyn (**u**) fluorescence intensity in the SNc. **v**, **w** Quantification of the percent area of the SNc occupied by NPTX1 + (**v**) and pSyn + (**w**) staining. Data expressed as mean fold change (± standard deviation) from PBS controls (analyzed with *t*-test with Welch’s correction). Scale bar in (**h**) is 250 μm and applies to (**a**–**h**), scale bar in (**p**) is 50 μm and applies to (**i**–**p**), scale bar in (**s**) is 10 μM and applies to (**q**–**s**)

We also evaluated changes in a representative complement regulator, for which we selected NPTX1, at the protein level using IF in midbrain tissue from PBS and α-syn PFF injected rats (Fig. 8). NPTX1 immunoreactivity was decreased in the ventral midbrain of α-syn PFF injected rats (Fig. 8e–h, m–p) compared to PBS controls (Fig. 8a–d, i–l). High magnification imaging of NPTX1 in the SNc of PBS controls revealed diffuse NPTX1 immunoreactivity within the somata of nigral neurons combined with abundant Nptx1 puncta studding the neuropil throughout the SN (Fig. 8i–l). In contrast, NPTX1 immunoreactivity was visibly decreased in both nigral neuron somata and the SNc neuropil in α-syn PFF injected rats (Fig. 8m–p). Nigral neurons that were largely devoid of pSyn + aggregates or contained smaller aggregates retained a strong, diffuse staining pattern that resembled NPTX1 immunoreactivity in nigral neurons of control animals (arrowheads in Fig. 8m–p). However, this diffuse staining pattern was almost entirely absent in neurons that contained pSyn + aggregates, particularly aggregates that appeared more compact and mature where bright NPTX1 puncta colocalized with pSyn (arrows in Fig. 8m–p and q–s). Quantification of NPTX1 fluorescence in the SNc confirmed a significant decrease in both NPTX1 IF intensity (Fig. 8t) and NPTX1 percent area staining (Fig. 8v) in α-syn PFF injected rats that was paralleled by significantly increased levels of pSyn in the SNc (Fig. 8u, w). These data suggest that, in addition to *Nptx1* gene downregulation, NPTX1 protein may also be sequestered into α-syn aggregates during the development of synucleinopathy, further impairing its ability to regulate complement.

**Fig. 9:**
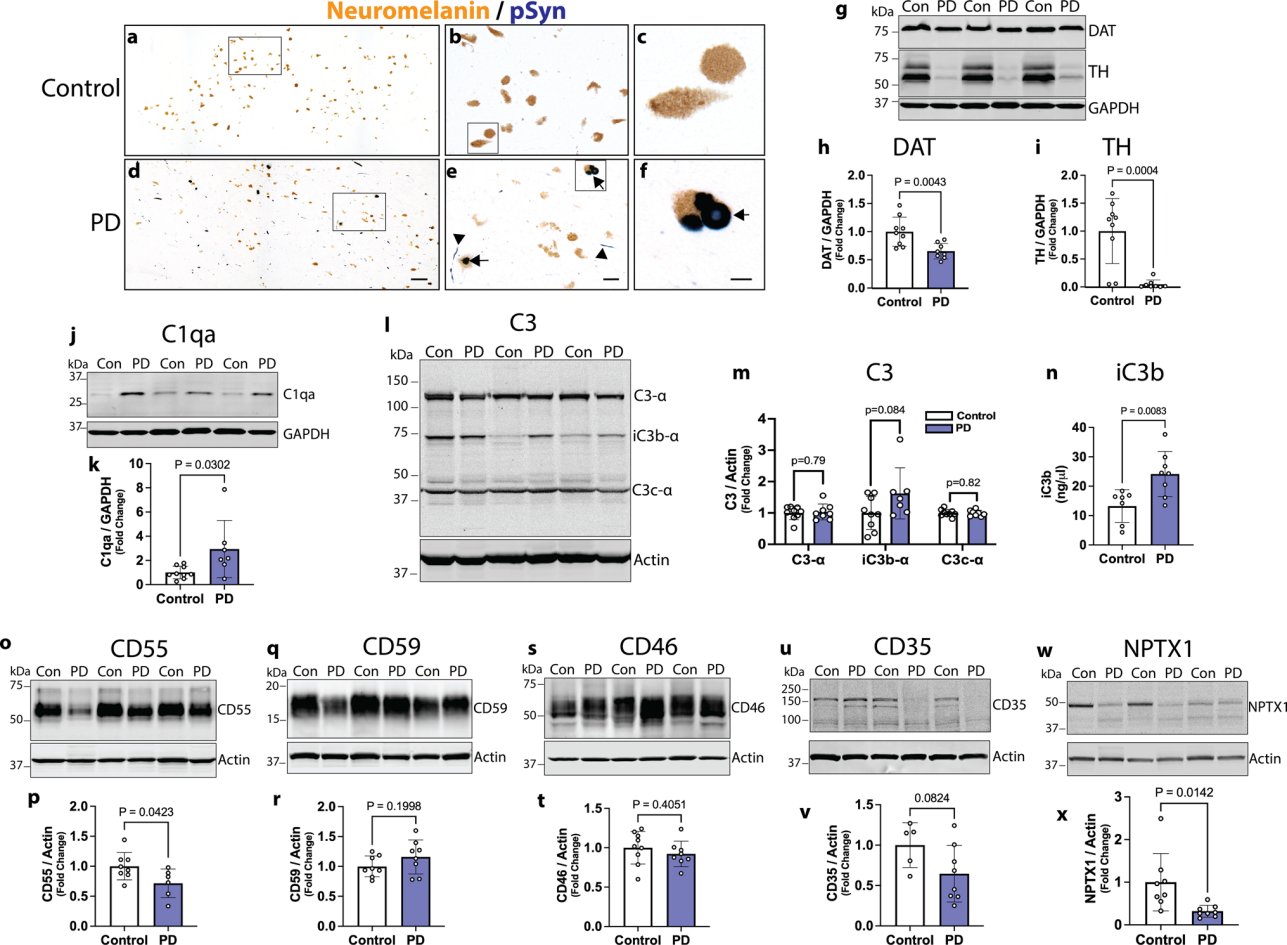
Expression of the complement regulatory proteins, CD55 and NPTX1, are decreased in the Parkinson’s disease substantia nigra. Postmortem human midbrain tissue from age matched controls and neuropathologically confirmed PD patients (*n* = 5–8/group) was obtained from the Michigan Brain Bank. **a**–**f** Immunostaining for serine 129 phosphorylated α-synuclein (pSyn) in the SNc of control (**a**–**c**) and PD (**d**–**f**) tissue. **b**–**e** High magnification images corresponding to the box in panels (**a**, **d**), respectively. **c**–**f** High magnification images corresponding to the box in panels (**b**, **e**)**,** respectively. Arrows in (**e**–**f**) indicate Lewy bodies while arrowheads in (**e**–**f**) indicate Lewy neurites. **g** Immunoblot of the dopamine transporter (DAT), tyrosine hydroxylase (TH) and glyceraldehyde 3-phosphate dehydrogenase (GAPDH) in control and PD SNc. **h**, **i**) Quantification of DAT (**h**) and TH (**i**) normalized to GAPDH. **j**, **k** Immunoblot of C1qa and GAPDH (**j**) and quantification of C1qa normalized to GAPDH from control and PD SNc (**k**). **l**, **m** Immunoblot of C3 and β-actin (**l**) and quantification of C3 α-chain (~ 115 kDa), iC3b α-chain (~ 67 kDa), and C3c α-chain (~ 40 kDa) normalized to β-actin from control and PD SNc (**m**). Data in (**h**, **I**, **k**, **m**) are expressed as mean fold change (± standard deviation) from controls and were analyzed by *t*-test with Welch’s correction. **n** Quantification of iC3b in control and PD SNc by sandwich ELISA. Data represent mean iC3b concentration (± standard deviation) analyzed by *t*-test with Welch’s correction. **o**–**x** Immunoblots and quantification of CD55 (**o**, **p**), CD59 (**q**, **r**), CD46 (**s**, **t**), CD35 (**u**, **v**), and neuronal pentraxin 1 (NPTX1; **w**, **x**) normalized to β-actin. Data expressed as mean fold change (± standard deviation) from controls and analyzed by *t* tests with Welch’s correction. Scale bar in (**d**) is 125 μm and applies to (**a**), scale bar in (**e**) is 25 μm and applies to (**b**), scale bar in (**f**) is 10 μm and applies to (**c**)

Decreased complement regulatory proteins (CD55, CD59 and NPTX1) combined with the increased complement activation observed in the SN of PFF-injected rats could render nigral dopamine neurons vulnerable to complement-mediated attack. Complement activation is reported in the PD brain [42, 87], however, prior reports did not specifically quantify complement regulatory proteins in PD-affected brain regions. Thus, we quantified complement regulatory proteins in postmortem midbrain tissue from neuropathologically confirmed control and PD brains (see Supplemental Table 1 Online Resources for neuropathologic and demographic data). We confirmed the presence of Lewy pathology in PD tissue using pSyn staining (Fig. 9d–f) and immunoblotted for the dopamine transporter (DAT) and tyrosine hydroxylase (TH) to confirm nigral neuron degeneration (Fig. 9g–i). We also quantified C1qa and C3/iC3b to determine the degree of complement upregulation/activation in the PD brain. Levels of C1qa were significantly increased in the SNc of PD brains (Fig. 9j, k). In contrast, there was no change in levels of intact C3 between the control and PD SNc (Fig. 9l, m). Quantification of iC3b by immunoblot trended (*p* = 0.08) towards an increase while iC3b quantified by sELISA was significantly increased (Fig. 9l–n). Levels of CD55 (Fig. 9o, p) and NPTX1 (Fig. 9w, x) protein were significantly decreased in the SNc of PD brains compared to controls but no other significant changes were observed in complement regulators, though CD35 trended towards a decrease (*p* = 0.08). These data, combined with the changes observed in the SNc of PFF injected animals, suggest that downregulation of CD55 and NPTX1 and increased levels of C1q and iC3b are central pathological changes associated with PD neuropathology.

**Fig. 10:**
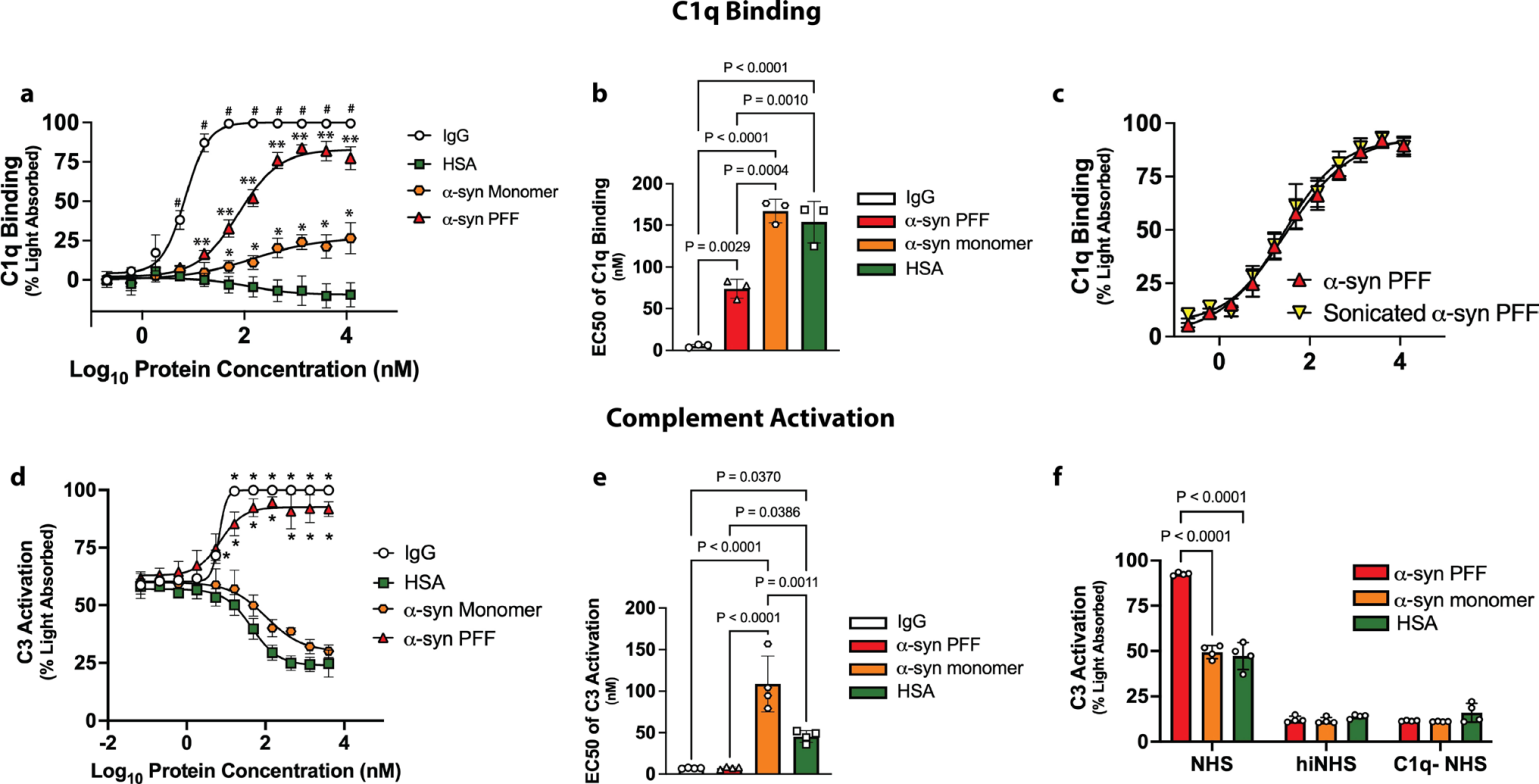
Aggregated α-synuclein activates the complement system in a C1q-dependent manner. **a** C1q binding to human IgG (positive control), human serum albumin (HSA, negative control), human α-synuclein (α-syn) monomers and human α-syn preformed fibrils (PFFs), expressed as mean percent light absorbed (± standard deviation, *n* = 3 biological replicates) analyzed by two-way ANOVA and Tukey’s multiple comparison. ^#^ significantly different from HSA, α-syn monomer and PFFs within same concentration; ** significantly different from HSA and α-syn monomer within same concentration; * significantly different from HSA within the same concentration. **b** C1q binding EC_50_ for human IgG, α-syn monomer, α-syn PFFs, and HSA, data shown as mean EC_50_ values (± standard deviation, *n* = 3 biological replicates) analyzed with a one-Way ANOVA and Tukey’s multiple comparison. **c** C1q binding to full length α-syn PFFs or sonicated α-syn PFFs, expressed as mean percent light absorbed (± standard deviation, *n* = 3 biological replicates) analyzed with a two-way ANOVA and Tukey’s multiple comparison. **d** Complement activation (indexed by formation of C3b/iC3b) by human IgG (positive control), HSA (negative control), human α-syn monomer and human α-syn PFFs, expressed as mean percent light absorbed (± standard deviation, *n* = 4 biological replicates) analyzed by two-way ANOVA and Tukey’s multiple comparison. * Significantly different from HSA and α-syn monomer within the same concentration. **e** EC_50_ for complement activation by human IgG, human α-syn monomer, human α-syn PFFs, and HSA, expressed as mean EC_50_ values (± standard deviation, 4 independent biological replicates) analyzed with a one-Way ANOVA and Tukey’s multiple comparison. **f** Complement activation assay using human α-syn monomer, human α-syn PFFs or HSA using normal human serum (*NHS*), heat inactivated NHS (*hiNHS*), or C1q depleted NHS (*C1q- NHS*), expressed as means percent light absorbed (± standard deviation, *n* = 4 biological replicates) analyzed by one-way ANOVA and Tukey’s multiple comparison

Finally, we developed in vitro assays to test whether pathological α-syn directly binds C1q and activates complement (Fig. 10). C1q bound to human IgG (positive control) and human α-syn PFFs in a concentration dependent- and saturable manner. C1q also bound weakly to human α-syn monomers at higher concentrations but did not bind the negative control, HSA (Fig. 10a). The average EC50 of C1q binding was 5.45 nM for human IgG, 73.68 nM for human α-syn PFFs, 167 nM for human α-syn monomer, and 153.8 nM for HSA (Fig. 10b). The EC50 of IgG binding to C1q was significantly lower than all other proteins, while the EC50 of α-syn PFF binding to C1q was significantly lower than HSA and α-syn monomers (Fig. 10b). There was no significant difference between the EC50 of α-syn monomer and the negative control, HSA, for C1q binding. Finally, to determine if the size of α-syn fibrils affects C1q binding, we performed the C1q binding assay with full length human α-syn PFFs and sonicated α-syn PFF, where both intact and sonicated human α-syn PFFs bound C1q in an identical manner (Fig. 10c).

We next sought to determine if aggregated α-syn activates the complement system in a cell-free system. Human IgG (positive control) and human α-syn PFFs increased the formation of C3b/iC3b (cleaved/activated forms of C3) in a concentration-dependent manner that was saturable. In contrast, human α-syn monomer and HSA did not increase C3b/iC3b levels (Fig. 10d). Instead, levels of activated C3 decreased at the higher concentrations of HSA and α-syn monomer compared to blank wells containing only blocking buffer (2% bovine serum albumin). The average EC50 for C3 activation was 6.9 nM for human IgG, 7.2 nM for human α-syn PFFs, 108.5 nM for human α-syn monomer, and 45.2 for HSA (Fig. 10e). The EC50 for IgG and human α-syn PFF induced C3 activation was significantly lower than human α-syn monomer and HSA, while the EC50 for C3 activation in the α-syn monomer group was significantly higher than all other groups (Fig. 10e). Finally, to determine if complement activation by pathological α-syn is mediated through the classical activation pathway, we repeated the complement activation assay using C1q depleted normal human serum (C1q- NHS). Depletion of C1q from serum completely prevented C3 activation in response to α-syn PFFs, down to the level of the negative control, heat inactivated NHS (hiNHS; Fig. 10f). Together, these data show that pathological α-syn can directly bind C1q and activate the complement system in a C1q-dependent.

## Discussion

Uncontrolled complement activation is increasingly recognized as a potential driver of neuroinflammation and neurodegeneration in PD, and previous studies report complement upregulation and activation in the PD brain and α-syn-based preclinical models of PD [6, 13, 28, 30, 33, 42, 43, 46, 57, 62, 75, 81, 87, 88, 94]. However, these studies have investigated complement activation at a time when significant neurodegeneration has already occurred. As such, it has been unclear whether complement activation is triggered directly by pathological α-syn. Thus, the goal of the current study was to determine if pathological α-syn activates the complement system prior to neurodegeneration.

### Pathological α-syn directly activates complement

One of the most salient conclusions from the current study is that aggregated forms of α-syn can directly activate the complement system. The current study and our recent report [75] uniquely demonstrate complement upregulation and activation during the early phases of synucleinopathy, indicating these changes occur in response to aggregated α-syn, independent of degeneration. Consistent with this interpretation, both C3 expression and levels of activated C3 are increased in the SNc of α-syn PFF injected rats during early synucleinopathy, where C3 upregulation significantly correlates with pSyn burden. Microglia expressing C3 and CR3 are increased in the SNc of α-syn PFF injected rats, where they localize to pSyn + inclusions and correlate with pSyn levels. Finally, C3 + microglia also spatially overlap with pSyn + aggregates in the Cx and ST and correlate with levels of pSyn in the Cx. The striking spatial correspondence between pSyn + aggregates and C3/CR3 + microglia, combined with the significant correlation between levels of C3 and pSyn load, strongly implicate complement upregulation in response to aggregated α-syn across affected brain regions. Importantly, the correlation between C3 and pSyn burden (in SNc and Cx) occurred in regions anatomically distal from the injection site (ST), suggesting the effects are driven by aggregates composed of endogenous misfolded α-syn, not local effects from exogenous protein delivery or injection-related injury. In contrast, the lack of correlation between C3 and α-syn pathology in the ST may reflect the presence of injection-related effects across groups. Consistent with this overall interpretation, despite increased levels of intact C3, levels of activated C3 were unchanged in the SN of α-syn monomer injected animals compared to PBS controls, further indicating that complement activation in SN is not driven by exposure to α-syn protein per se, but rather by the ensuing development of synucleinopathy.

The elevation of intact (i.e., un-activated) C3 in the SN of α-syn monomer injected animals was significantly lower compared to α-syn PFF-injected animals and was not associated with a significant increase in activated C3. These data suggest that exposure to exogenous α-syn protein may contribute to modest C3 upregulation without activation. Despite our efforts to confirm the soluble state of α-syn monomer preparations during production and purification, one possible explanation for C3 upregulation in monomer injected animals is the unintended presence of low amounts of α-syn aggregates that may have formed prior to- or during injections. Indeed, we and others have observed de novo aggregation of α-syn under standard handling conditions in the absence of intentional aggregation inducers (i.e. agitation at 37 °C). Thus, although monomeric α-syn modestly increased intact C3 levels in the SN, these findings should be interpreted cautiously and future experiments incorporating stringent controls for spontaneous α-syn aggregation are needed to determine if monomeric α-syn is sufficient to drive C3 upregulation.

C3 activation in response to aggregated α-syn, but not monomeric α-syn, is corroborated by direct evidence from our in vitro complement assays. In these assays, α-syn aggregates bound C1q and induced a concentration-dependent deposition of activated C3, while α-syn monomers did not. These experiments confirm aggregated α-syn, but not α-syn monomer, is sufficient to activate the complement system, which parallels in vivo data comparing α-syn monomer and PFF injected animals. These findings align with the fact that monomeric α-syn and complement components, including C1q, coexist in many body tissues, including blood and brain [2, 29]. Accordingly, if monomeric α-syn could activate complement, one would expect a basal level of complement activation in healthy individuals. However, this is not the case, as chronic complement activation is typically associated with disease and/or inflammatory disorders.

Results from our in vitro complement activation assays are consistent with some previous findings but not others [20, 36]. For example, [36] found that full length, monomeric α-syn did not activate the complement system, whereas the alternatively spliced isoform, α-syn 112, did. In contrast, [20] found that full length α-syn, but not the truncated α-syn 1–95, induced complement activation. Our results align closely with those of [36], however, the reason for these discrepancies is unclear. As noted above, one possibility is that some complement activation attributed to monomeric α-syn in previous studies may be the result of de novo aggregate formation. This hypothesis is in line with the increased complement activation reported with α-syn 112, an isoform with accelerated aggregation kinetics [44, 63]. However, it would not account for the lack of complement activation reported with α-syn 1–95, as C-terminal truncation of α-syn also accelerates aggregation [71]. Alternatively, methodological differences, such as the complement activation product quantified (C3b/iC3b versus C4b versus C5b-9), may explain these discrepant findings. Future studies that rigorously control for de novo aggregate formation and index activation of multiple complement targets are needed to definitively determine whether monomeric α-syn can activate the complement system.

Finally, while some indices of complement activation were increased in postmortem SNc tissue, these increases were modest when compared to α-syn PFF injected rats. The current study used late-stage postmortem PD tissue to validate changes observed during early synucleinopathy in α-syn PFF injected rats. This temporal disconnect prevents a direct comparison to the early complement changes observed in preclinical models, however it may yield important insights into the time course of complement activation in the context of synucleinopathy. For example, we detected robust upregulation of C3 and increased levels of iC3b in α-syn PFF injected rats. In contrast, levels of intact C3 were not changed in the PD SNc, while levels of iC3b were significantly increased when quantified by sELISA. These differences support the hypothesis that C3 upregulation and activation are early responses to synucleinopathy. The physical association between C3/CR3 expressing microglia with pSyn + neurons during the early phases of pathology suggest that reactive glia may use complement to target synucleinopathy affected neurons. Subsequently, results from human tissue indicate complement activation and expression diminish after significant neurodegeneration occurs. These comparative findings suggest that some aspects of the complement response to synucleinopathy are stage dependent.

### Synucleinopathy activates complement primarily through the classical pathway

Data from the current study indicate pathological α-syn stimulates complement activation mainly through the classical pathway. We found significant upregulation of targets from both the classical and alternative pathways, but the magnitude of classical pathway gene upregulation was greatest. Consistent with classical pathway engagement, purified human C1q bound aggregated human α-syn and depletion of C1q from human serum completely abolished complement activation in response to pathological α-syn. However, the upregulation of alternative pathway components, such as Factor D and Factor B, suggests the alternative pathway may amplify the complement response to synucleinopathy once initiated through C1q. Together, these findings indicate synucleinopathy drives a broad elevation in complement gene expression, with a pronounced bias toward classical pathway activation.

The pattern of classical pathway upregulation and activation observed in α-syn PFF injected rats aligns with complement activation in the PD brain. Numerous studies utilizing a variety of methods (including gene expression, transcriptomics, proteomics, and histology) document upregulation and activation of classical pathway targets in postmortem PD tissue [13, 30, 42, 46, 87]. Among these altered complement targets, C4 and C1-complex proteins are the most consistently elevated and/or activated, mirroring findings from the present study. Additionally, levels of activated C4 are increased in the brains of DLB patients [28, 81], indicating complement activation is not unique to PD but a more general response to synucleinopathy. Finally, our results align with prior work demonstrating that C1q is required for α-syn-induced complement activation [20]. These data indicate complement activation is a common response to aggregated α-syn, and support the conclusion that C1q recognizes pathological α-syn as a damage associated molecular pattern (DAMP) or a neurodegeneration associated molecular pattern (NAMP), thereby triggering complement activation through the classical pathway.

### Early synucleinopathy stimulates phagocytosis and anaphylatoxin signaling

Following activation, data herein indicate opsonization and anaphylatoxin signaling are the primary complement effector responses enacted during the early phases of synucleinopathy. Complement activation ultimately results in the cleavage of C3 into a small peptide named C3a and a larger fragment named C3b. C3a is a potent anaphylatoxin that increases local inflammation by recruiting and activating immune cells [8, 26, 45, 80] C3b is an opsonin that covalently binds targets to mark them for phagocytic clearance by cells expressing CR1 [15, 49]. However, C3b is rapidly cleaved by Factor I and Factor H to generate iC3b, which is also a functional opsonin that recruits phagocytes through interaction with CR3 and CR4 [1, 64].

In the current study, levels of the opsonin, iC3b, were significantly increased in both the ST and SNc of α-syn PFF injected rats. Additionally, expression of the *Itgam* gene, which encodes a subunit of CR3, was one of the most highly upregulated receptor genes in the SNc of α-syn PFF injected rats. Like C3 + microglia, microglia expressing CR3 were increased and localized near pSyn + neurons in the SNc. CR3 is also a pattern recognition receptor that can directly bind α-syn, thereby facilitating microglial uptake, and genetic deletion of CR3 prevents microglial activation in response to pathological α-syn [25, 32, 83, 90]. These data strongly implicate CR3 and complement mediated phagocytosis in the response to synucleinopathy.

It is currently unclear if α-syn aggregates or neurons containing these aggregates are opsonized or targeted by CR3-expressing phagocytes in α-syn PFF injected rats. Complement-expressing microglia (C3 + and CR3 +) appeared to be extending processes around neurons containing α-syn aggregates, suggesting they may be targeting synucleinopathy-affected neurons for phagocytic clearance. However, we did not observe colocalization of C3 with pSyn + aggregates at the time point investigated. The antibody used for immunoflourescent detection of C3 in the current study was not specific to activated forms of C3. Thus, it is possible that α-syn aggregates or the neurons containing aggregates were labeled with C3b/iC3b but were not detected because the reagents used were not specifically sensitive to activated C3. Alternatively, complement opsonins may label α-syn aggregates or nigral neurons at later timepoints in the degenerative cascade. Supporting this conclusion, remaining neuromelanin neurons and Lewy bodies label with iC3b in postmortem PD tissue [42]. Additional experiments are needed to determine if α-syn aggregates are opsonized during the early phases of synucleinopathy.

The current study also implicates anaphylatoxin signaling in the complement response to synucleinopathy. The anaphylatoxins, C3a and C5a, are potent inflammatory signaling molecules that function through interactions with their cognate receptors, C3aR and C5aR. C3aR and C5aR are expressed on multiple CNS cell types, most prominently microglia, but also astrocytes, endothelial cells, perivascular macrophages, and under specific conditions, neurons [61]. Activation of these receptors induces a wide range of proinflammatory responses, including glial priming and activation, chemotaxis, cytokine release, vascular changes and increased phagocytosis, while activation of neuronally expressed anaphylatoxin receptors is directly toxic to cultured neurons, including mesencephalic dopamine neurons [24, 60, 84]. *C3aR* and *C5aR* expression were significantly increased in the SNc of α-syn PFF-injected rats compared to controls, indicating increased C3a/C5a signaling during the early phases of synucleinopathy. C3aR upregulation is reported in the SN of A53T α-syn transgenic mice, AAV-α-syn injected mice and rotenone treated mice [6, 41, 91]. Our findings extend these observations by demonstrating anaphylatoxin receptor upregulation prior to degeneration in a mechanistically distinct model of α-syn pathology. Finally, we recently identified a distinct suite of immune genes upregulated in reactive microglia and astrocytes in the SN during the early phases of synucleinopathy [57, 75]. Interestingly, many of these genes or their associated networks overlap with anaphylatoxin-responsive transcriptional programs described in the context of AD [19, 40, 66], raising the possibility that C3a/C5a signaling regulates the conversion of homeostatic glia to the reactive state in PD. This convergence suggests anaphylatoxin receptor signaling may represent a shared inflammatory pathway across neurodegenerative diseases and highlights their potential as therapeutic targets in synucleinopathies.

### Complement dysregulation during early synucleinopathy

Complement regulators are a diverse group of proteins that restrain complement activation at different levels of the cascade to protect self-cells from excessive inflammation, opsonization and membrane attack. Complement regulators fall broadly into three categories, fluid-phase regulators, membrane bound regulators, and C1q binding proteins. In the current study we observed a consistent pattern of dysregulation, characterized by upregulation of fluid phase regulators (e.g. *C1Inh*, *Cfh*, *Clu*), paralleled by a downregulation of specific membrane bound regulators (e.g. *Cd55*, *Cd59*) and C1q-binding proteins (e.g. *Nptx1*, *Nptxr*). This imbalance suggests that key homeostatic “checkpoints” may be compromised in early synucleinopathy, potentially lowering the threshold for complement-mediated glial activation and/or neuronal injury.

Consistent with our recent finding that *C1 Inh* is increased in the SN of α-syn PFF injected rats [75], expression of *Cfh* and *Clu* were also significantly increased, indicating a non-cell autonomous upregulation of fluid phase regulators during early synucleinopathy. As α-syn directly activated the classical pathway in a C1q-dependent manner, increasing opsonin deposition and phagocytic receptor expression, increased C1 Inh and Cfh likely represent compensatory responses aimed at limiting mistargeted phagocytosis during heightened complement activation. Further, Clu upregulation may serve a dual role in the response to synucleinopathy, as Clu inhibits MAC formation but also acts as a molecular chaperone to prevent aggregation and promote clearance of misfolded proteins [5]. Thus, Clu upregulation in α-syn PFF injected rats may also be an attempt to mitigate α-syn aggregation.

Membrane-bound complement regulators are cell-surface proteins that protect host cells from unintended complement-mediated damage by locally inhibiting different steps of the complement cascade. In the current study, *Cd55* and *Cd59* expression were significantly decreased in the SN of α-syn PFF-injected rats during early synucleinopathy, and CD55 was also significantly decreased in the PD SNc. These results align with prior reports of CD55 downregulation in the PD SNc [33] and in the ST of α-syn PFF injected mice [88], Additionally, CD55 protein is decreased in the CSF of PD patients, where levels correlate with motor and cognitive impairment and help distinguish PD from controls [4]. Finally, few studies have linked CD59 to PD. One study found increased CD59 protein in the PD SNc affected by both Lewy pathology and neuronal loss compared with SNc affected by neuronal loss alone [62]. Collectively, these data support CD55 dysfunction as a central event in PD pathobiology and extend previous findings by demonstrating CD55 and CD59 downregulation during early synucleinopathy, suggesting complement dysregulation may be a durable change that contributes to degeneration.

Lastly, the current results also implicate dysregulation of C1q-binding proteins in the response to synucleinopathy. The neuronal pentraxins have recently emerged as important modulators of complement-mediated synaptic pruning in the CNS through their ability to bind C1q and inhibit classical pathway activation [37, 93]. Decreased nigral expression of NPTX1 during early synucleinopathy is a novel finding. However, previous reports found that NPTX2 accumulates in the PD SNc, where it colocalizes with α-syn in Lewy bodies and neurites [47], mirroring the colocalization of NPTX1 with pSyn aggregates in the SNc of α-syn PFF-injected rats in the current study. All neuronal pentraxins are decreased in the CSF of patients with PD, MSA and progressive supranuclear palsy [51]. Importantly, CSF neuronal pentraxin levels correlate with cognitive impairment, dopaminergic degeneration and motor impairment in PD, implicating the neuronal pentraxins in disease pathophysiology and as biomarkers [51].

Collectively, previous findings and current results implicate dysfunction or downregulation of multiple classes of complement regulators in the response to PD pathology. This supports a model in which complement dysregulation, driven both by increased activation and impaired regulation, contributes to neurodegeneration in synucleinopathies. Accordingly, therapeutic strategies aimed at maintaining or restoring complement regulator function could provide neuroprotection in PD and other synucleinopathies.

### Limitations of the current study

There are several limitations to the current study that should be considered when interpreting results. First, we were unable to comprehensively profile all components of the complement system. The complement system comprises over 50 proteins, and due to the targeted nature of the investigation, it was not feasible to profile potential changes in every component. Additionally, we could not localize the cell source of all differentially expressed complement components in α-syn PFF injected rats. Determining the cellular source of these changes could be critical for understanding how complement dysregulation contributes to neurodegeneration in synucleinopathies and future studies should be performed to address this issue. Lastly, the current study used late-stage postmortem PD tissue to validate molecular changes identified during the early phases of synucleinopathy in α-syn PFF-injected rats. As such, the molecular changes observed in postmortem tissue could reflect late-stage consequences of neurodegeneration, rather than the primary response to synucleinopathy. This temporal disconnect prevents a direct comparison to the early complement changes observed in preclinical models.

## Conclusions

We conclude that synucleinopathy stimulates complement upregulation and activation through the classical and alternative pathways, and causes complement dysregulation prior to overt degeneration. We determined that microglia are the primary cellular source of complement C3 in the rat brain during early synucleinopathy, and microglia upregulate C3 across multiple brain regions affected by synucleinopathy. Increased opsonization, phagocytosis and anaphylatoxin signaling are the primary complement effector responses initiated during early synucleinopathy. Further, early synucleinopathy causes downregulation of CD55, CD59 and NPTX1 in the SNc of α-syn PFF injected rats. CD55 and NPTX1 are also decreased in the PD SNc, indicating that complement dysregulation persists through the end stages of disease. Finally, aggregated α-syn, but not monomeric α-syn, directly activates the classical complement system in a C1q-dependent fashion. The robust complement activation and regulator dysfunction observed during the early phases of synucleinopathy reported herein could render neurons susceptible to complement-mediated attack and suggests restoring normal complement regulation could be a viable therapeutic strategy for synucleinopathies.

## Supplementary Information

Below is the link to the electronic supplementary material.

Supplementary file1 (PDF 8894 kb)

Supplementary file2 (XLSX 10 kb)

Supplementary file3 (XLSX 9 kb)

Supplementary file4 (XLSX 11 kb)

Supplementary file5 (XLSX 53 kb)

Supplementary file6 (XLSX 35 kb)

## Data Availability

The datasets generated and analyzed in the current study will be made available upon reasonable request to the corresponding author.

## Abbreviations

α-syn: Alpha-synuclein
AAALAC: Association for Assessment and Accreditation of Laboratory Animal Care
C3bBb: Alternative pathway C3 convertase
BSA: Bovine serum albumin
CPN: Carboxypeptidase-N
C1-INH: C1 inhibitor
C4b2a: Classical/lectin pathway C3 convertase
CLU/Clu: Clusterin
C3: Complement component 3
C5: Complement component 5
Cfb: Complement factor B
Cfd: Complement factor D
Cfh: Complement factor H
Cr1l: Complement receptor 1-like protein
CR3: Complement receptor 3
C4BP: C4 binding protein
DLB: Dementia with Lewy body
DAT: Dopamine transporter
ddPCR: Droplet digital PCR
dPBS: Dulbecco’s phosphate-buffered saline
EM: Electron microscopy
EDTA: Ethylenediaminetetraacetic acid
FH: Factor H
FI: Factor I
GAPDH: Glyceraldehyde-3-phosphate dehydrogenase
hiNHS: Heat-inactivated normal human serum
IACUC: Institutional Animal Care and Use Committee
IPTG: Isopropyl β-D-1-thiogalactopyranoside
MBL: Mannose-binding lectin
Mbl2: Mannose-binding lectin 2
Masp1: Mannan-binding lectin serine protease 1
MAC: Membrane attack complex
MSA: Multiple system atrophy
Nptx1: Neuronal pentraxin 1
Nptx2: Neuronal pentraxin 2
Nptxr: Neuronal pentraxin receptor
NPTXs: Neuronal pentraxins
NHS: Normal human serum
PD: Parkinson’s disease
PBS: Phosphate-buffered saline
PBS-T: Phosphate-buffered saline with Tween-20
pSyn: Serine 129 phosphorylated alpha-synuclein
PFFs: Pre-formed fibrils
Rpl13: Ribosomal protein L13
sELISA: Sandwich enzyme-linked immunosorbent assay
SDS: Sodium dodecyl sulfate
ST: Striatum
SN: Substantia nigra
SNc: Substantia nigra pars compacta
SNr: Substantia nigra pars reticulata
ThT: Thioflavin-T
TBS: Tris-buffered saline
TBS-Tx: Tris-buffered saline with Triton X-100
TBS-T: Tris-buffered saline with Tween-20
TH: Tyrosine hydroxylase
VTN: Vitronectin
WT: Wild type
