# Supplementary material for: Complement dysregulation during the early phases of synucleinopathy
Source: Acta Neuropathol. 2026 Jul 20;152(1):7. doi: 10.1007/s00401-026-03057-8 (PMC13385250; doi:10.1007/s00401-026-03057-8)
Supplement: Supplementary file 1 — Supplementary file1 (PDF 8894 kb) [file 401_2026_3057_MOESM1_ESM.pdf]

Supplementary material for *Acta Neuropathologica* manuscript:

Complement Dysregulation During the Early Phases of Synucleinopathy

Hina Khan[1], Mary Gifford[2], Arash Kordbacheh[3], Asher Bury, Spencer Panousheck[1,] Allyson Cole-Strauss[1], Christopher J. Kemp[1], Kelvin C. Luk[4], Kathy Steece-Collier[1], Nathan C. Kuhn[1], Nicholas M. Kanaan[1], Caryl E. Sortwell[1], Joseph R. Patterson[1], Matthew J. Benskey[1]

[1] Department of Translational Neuroscience, Michigan State University, Grand Rapids, MI USA
[2] Cell and Molecular Biology Program, Grand Valley State University, Allendale, MI USA
[3] Biomedical Sciences Program, Grand Valley State University, Allendale, MI USA
[4] Center for Neurodegenerative Disease Research, Department of Pathology and Laboratory Medicine, University of Pennsylvania Perelman School of Medicine, Philadelphia, PA USA

Corresponding Author:
Matthew J. Benskey, Ph.D.
Department of Translational Neuroscience
Michigan State University
Grand Rapids, MI 49503
benskeym@msu.edu

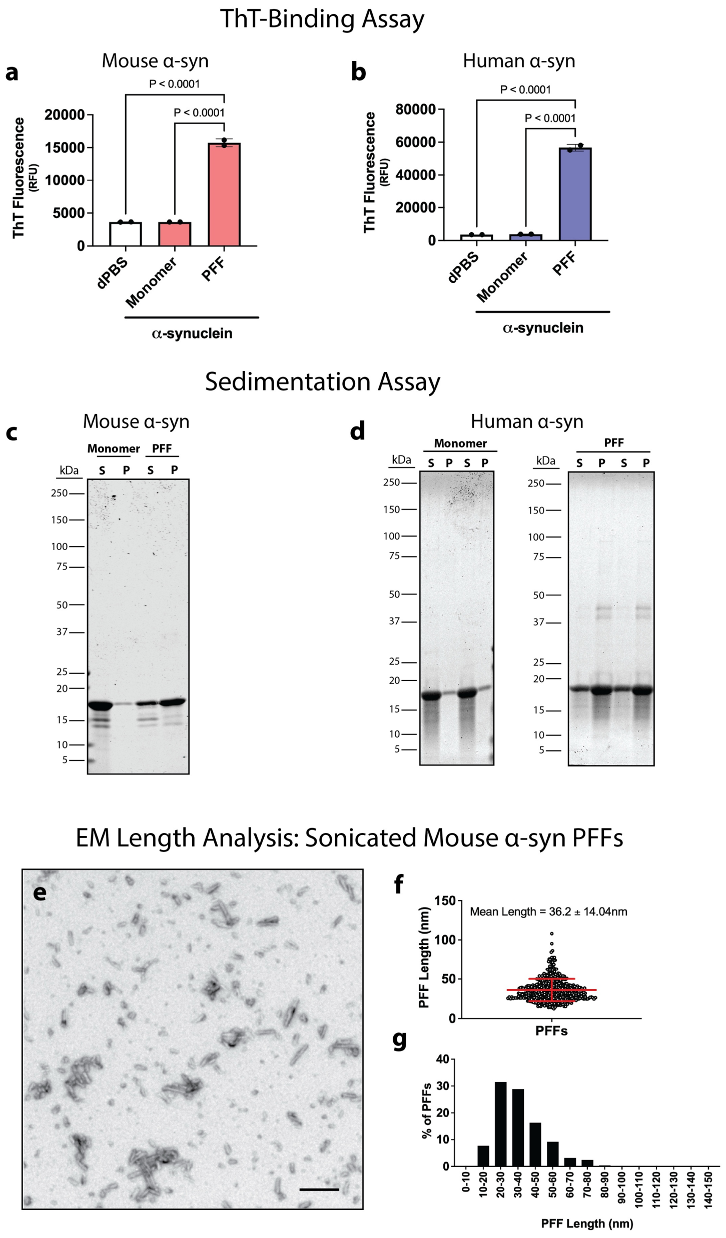

Supplemental Fig. 1: α-syn Preformed Fibril Quality Control. Quality control was performed on recombinant, full length mouse and human α-synuclein (α-syn) pre-formed fibrils (PFFs). **a-b**) β-sheet amyloid structures in assembled recombinant full length α-syn PFFs, α-syn monomers and Dulbecco's phosphate buffered saline (dPBS) vehicle were quantified with Thioflavin T (ThT). Recombinant mouse α-syn PFFs (**a**) and human PFFs (**b**) bound significantly more ThT than α-syn monomers and dPBS vehicle control (n=2 replicates, data analyzed by one way ANOVA with Tukey's multiple comparison test). **c-d**) A sedimentation assay was performed to detect high molecular weight α-syn species. Mouse (**c**) and human (**d**) α-syn monomers resolved primarily in the soluble fraction (S) while the majority of α-syn PFF protein resolved in the pellet fraction (P). **e-f)** Quantification of sonicated mouse α-syn PFF length by electron microscopy (EM). **e**) Representative electron micrograph showing negatively stained, sonicated mouse α-syn PFFs used for stereotactic injection. **f**) Size (nm) distribution of sonicated α-syn PFFs quantified from EM images. **g**) Size distribution (nm) of sonicated mouse α-syn PFFs expressed as a percentage of total PFFs that fall into 10nm bins. The mean length of sonicated moues α-syn PFFs was 36 ± 14nm and > 93% of all fibrils measured were ≤ 50nm. Scale bar in panel (**e**) is 200nm.

a

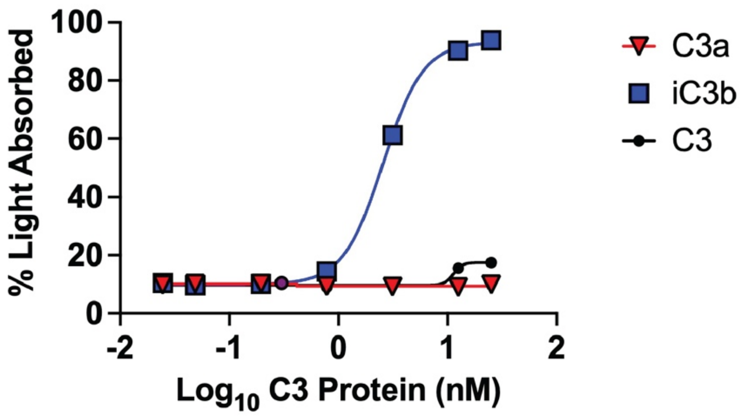

b

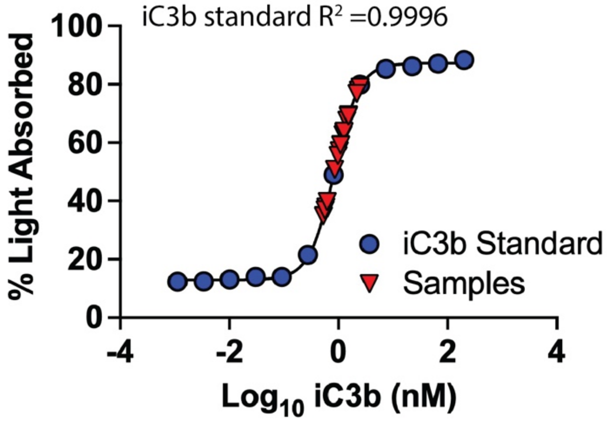

Supplemental Fig 2: Human iC3b Sandwich ELISA. a) The specificity of the assay for human iC3b was validated using purified human C3 (black lines with circles), human iC3b (blue lines with squares) and human C3a (red lines with triangles) ranging from 50nM to 0.048nM. **b**) Plot of iC3b standards (blue circles) and human brain lysate samples (red triangles) that were used to quantify iC3b in the substantia nigra of control and PD brains (these samples and standards correspond to **Figure 9n**). Note that the human samples are within the linear range of the standards.

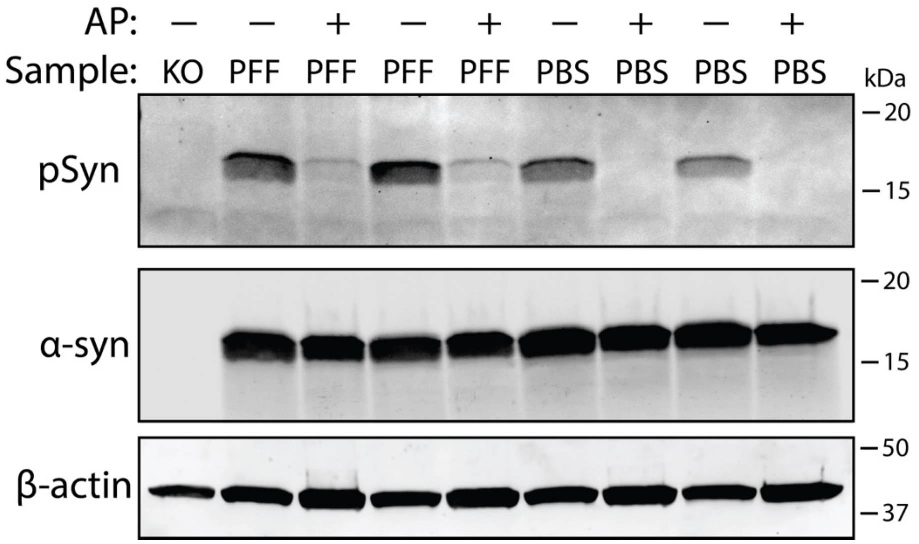

Supplemental Fig 3. Testing the Specificity of the Antibody Used to Biochemically Quantify pSyn. Striatal lysates from α-synuclein (α-syn) pre-formed fibril (PFF) or phosphate buffered saline (PBS) injected rats (n=4-5/group; 2-months post-injection), and untreated α-syn germline knockout mice (KO; 6 months of age, n=3) were incubated with (+) or without (-) alkaline phosphatase (AP) to dephosphorylate α-syn. Blots were probed with a pan α-synuclein antibody (α-syn) or a phospho-serine 129 specific α-syn (pSyn) antibody. Beta-actin was used as a loading control. Neither antibody detected protein bands in α-syn KO lysates. The pSyn antibody detected a protein band resolving at ~17kDa, which was increased in the ST of α-syn PFF injected rats compared to PBS controls. De-phosphorylation of lysates eliminated pSyn antibody signal in ST lysates of PBS treated rats and robustly decreased detection in PFF animals.

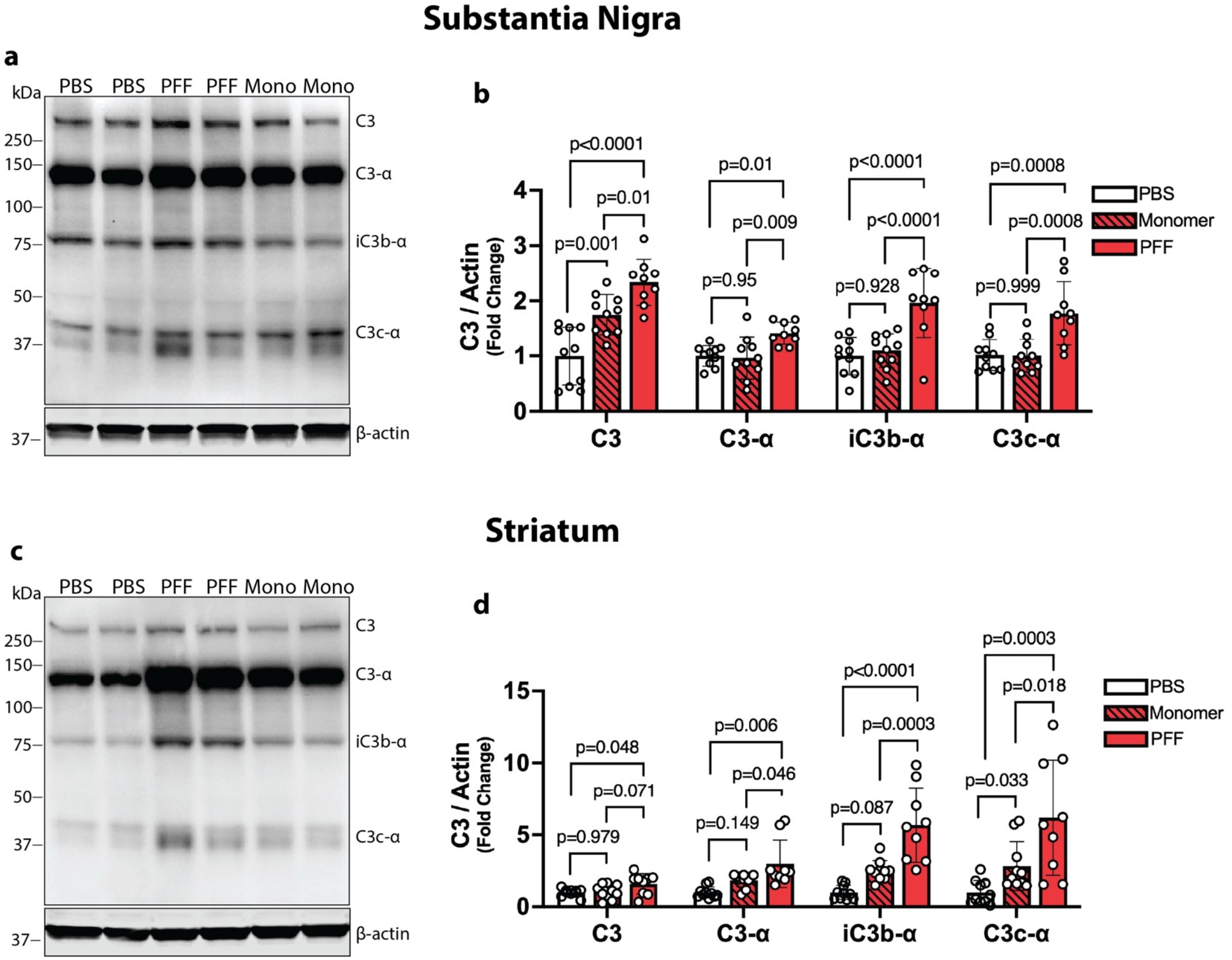

Supplemental Fig 4. α-synuclein PFFs Robustly Activate Complement Component 3 Compared to α-synuclein Monomers *In Vivo*. Rats (n=4-5 sex/group) received intra-striatal injections of mouse α-synuclein (α-syn) preformed fibrils (PFFs), an equal amount of mouse α-syn monomer or an equal volume of phosphate buffer saline (PBS) and were euthanized 2-months post-injection. No significant sex differences were detected within any group (see **Supplementary Table 5** for data on individual sexes), thus sexes were combined within groups for all analyses. **a, c**) Representative immunoblot of C3 and β-actin from the ipsilateral substantia nigra (SN; **panel a**) or the ipsilateral striatum (ST; **panel c**). **b, d**) Quantification of C3 whole molecule (~190 kDa), C3 α-chain (~115 kDa), iC3b α-chain (~75 kDa), and C3c α-chain (~34kDa) normalized to β-actin in the SN (**b**) and ST (**d**). Data analyzed with a one-way ANOVA and Tukey's multiple comparison test. Data are group means ± standard deviation expressed a fold change from PBS group.

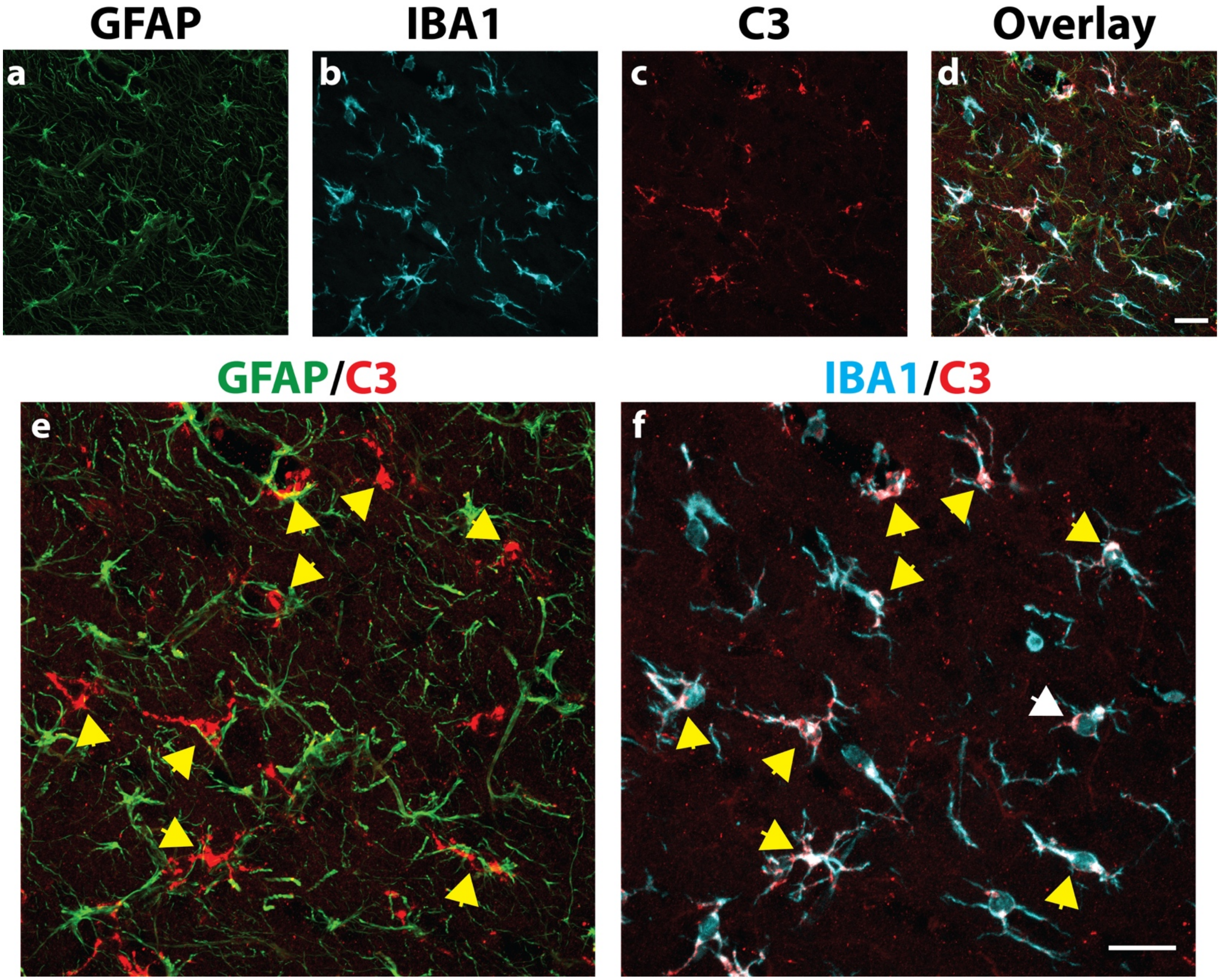

Supplemental Fig. 5: Microglia are the Primary Cellular Source of Complement C3 in the Rat Brain. Tissue from the ipsilateral cortex of α-synuclein (α-syn) pre-formed fibril (PFF) injected rats was processed for immunofluorescent (IF) detection of astrocytes (**panel a**; glial fibrillary acidic protein, GFAP; green), microglia (**panel b**; ionized calcium binding adaptor molecule 1, IBA1; cyan) and complement component 3 (**panel c**; C3; red). Enlarged overlay images of GFAP and C3, or IBA1 and C3 are shown in panels (**e**) and (**f**), respectively. Arrows in (**f**) show colocalization of C3 and IBA1 signal. Scale bars in (**d)** and (**f)** are 25μm and apply to (**a-c)** and **(e)** , respectively.

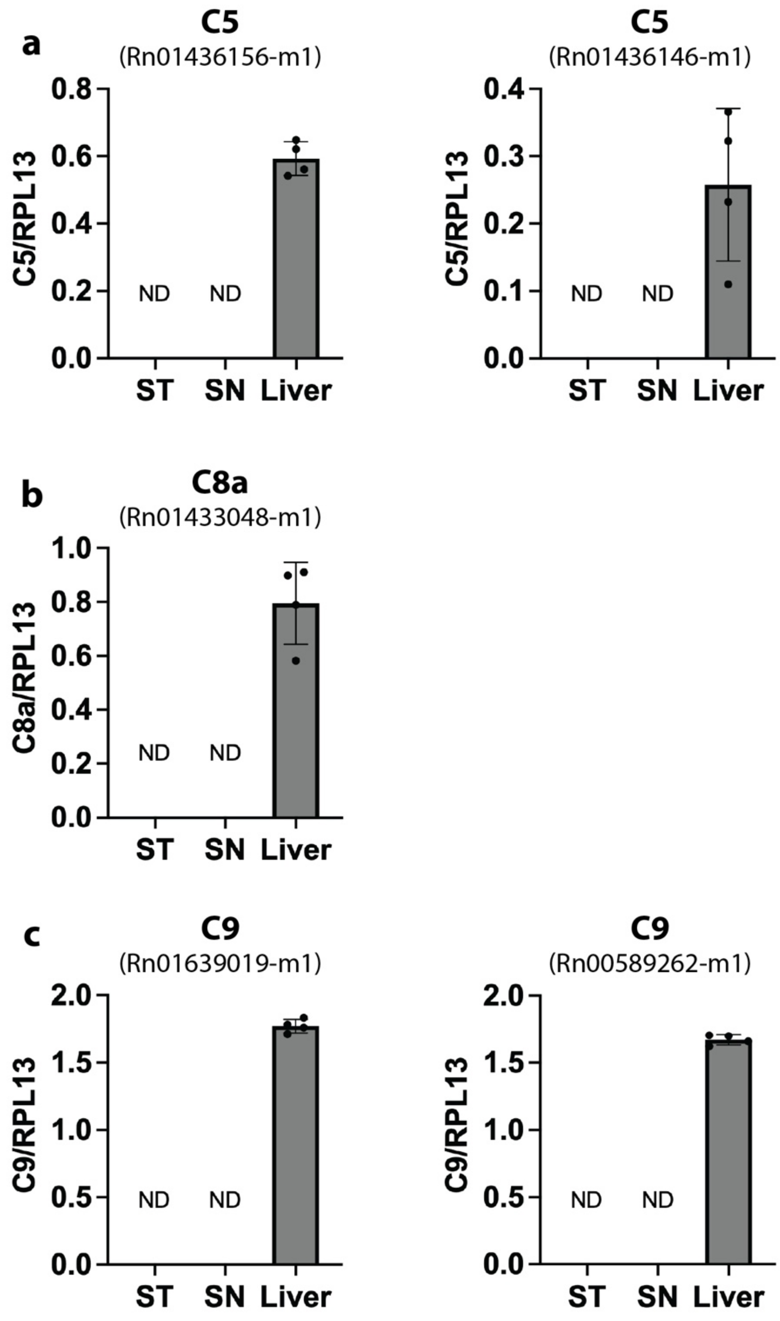

Supplemental Fig. 6: Expression of Genes Encoding the Terminal Complement Pathway Targets, C5, C8a and C9, in the Striatum and Substantia Nigra of α-syn PFF Injected Rats. Male and female rats received intra-striatal injections of α-synuclein (α-syn) pre-formed fibrils (PFFs) (n=4-5/group) and were euthanized 2-months post-injection **a-c**) The liver, ipsilateral substantia nigra (SN) and ipsilateral striatum (ST) from α-syn PFF injected animals were analyzed for transcripts representing different targets in the terminal complement pathway using droplet digital PCR. Quantification of complement *C5* (**a**), *C8a* (**b**), and *C9* (**c**) in the ST, SN and liver of PFF injected rats. Data represent the mean target gene levels ($\pm$ standard deviation) normalized to a reference gene, ribosomal protein L13 (Rpl13). Catalogue numbers of the specific primer/probes sets are noted above each respective histogram. Not detected (ND).

a)

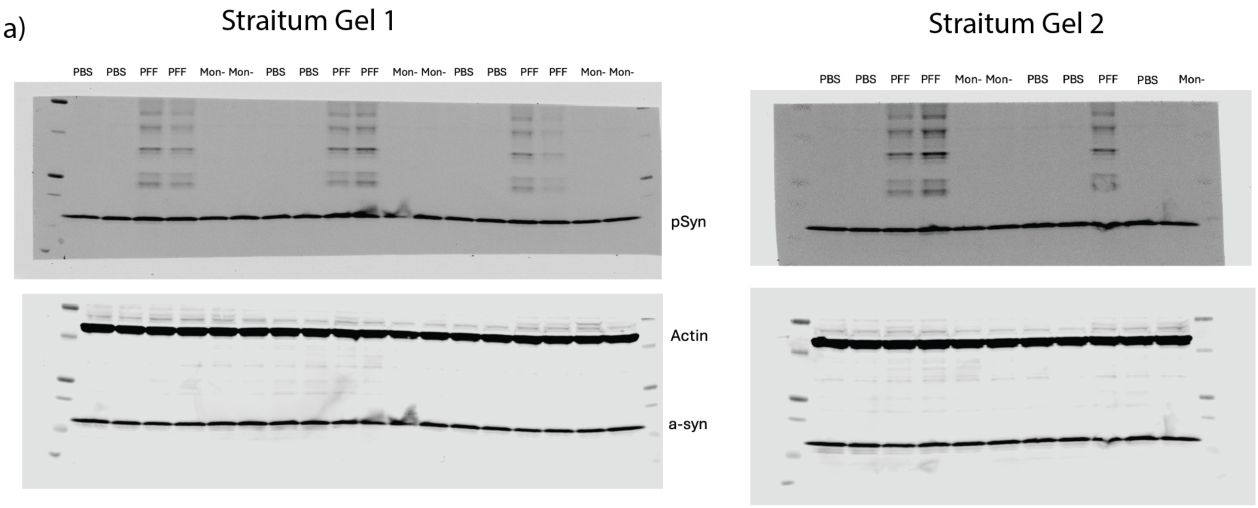

b)

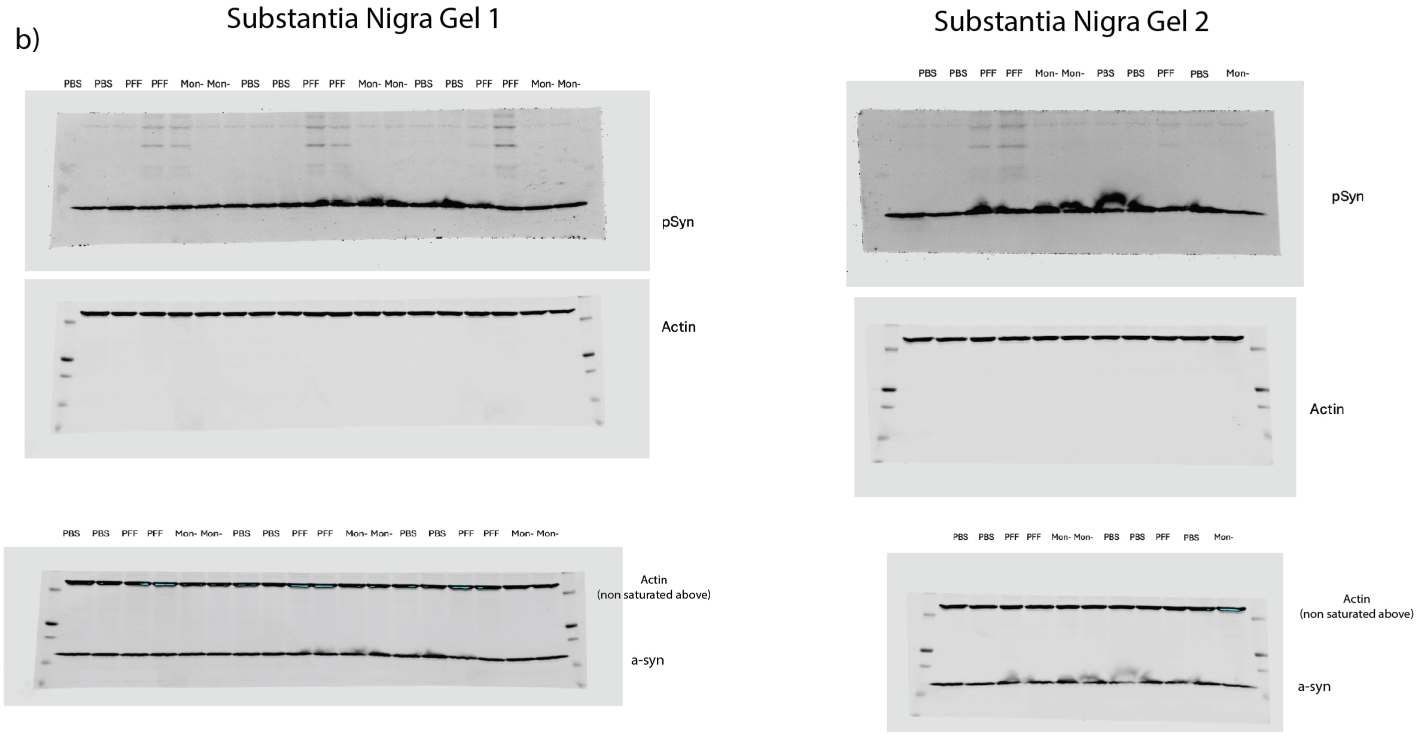

Supplemental Fig. 7: Uncropped Western blots corresponding to Figure 2 in main manuscript, showing serine 129 phosphorylated α-synuclein (pSyn), α-synuclein (α-syn) and β-actin loading control in the Striatum (a) and substantia nigra (b) of PBS and α-syn PFF injected rats.

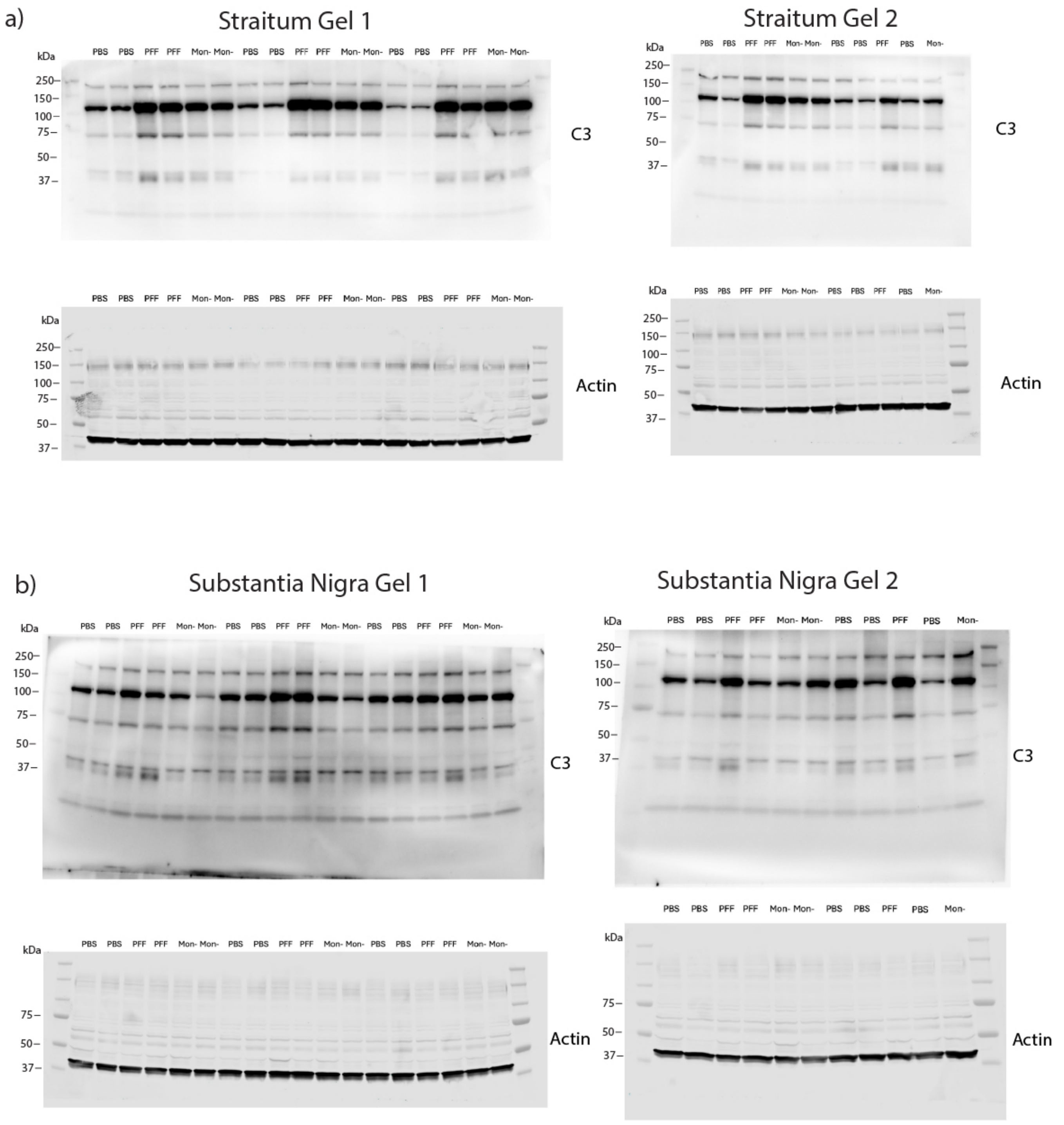

Supplemental Fig. 8: Uncropped Western blots corresponding to Figure 2 in main manuscript and supplemental figure 4, showing complement C3 and β-actin loading control in the Striatum (a) and substantia nigra (b) of PBS and α-syn PFF injected rats.

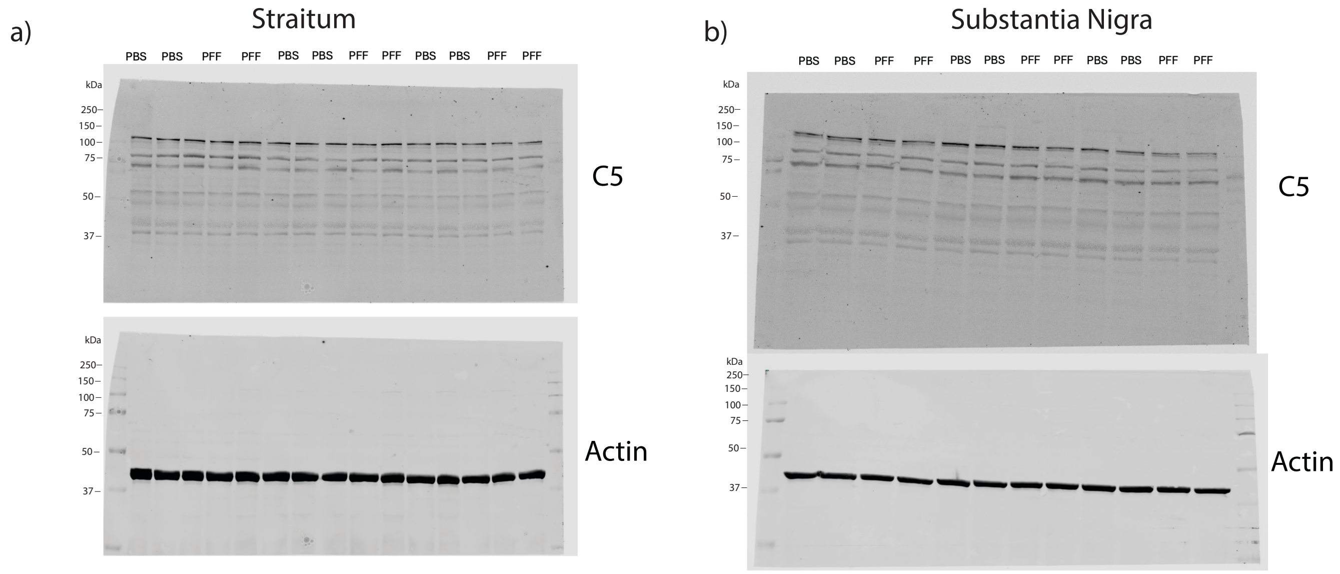

Supplemental Fig. 9: Uncropped Western blots corresponding to Figure 5 in main manuscript, showing complement C5 and β-actin loading control in the striatum (a) and substantia nigra (b) of PBS and α-syn PFF injected rats.

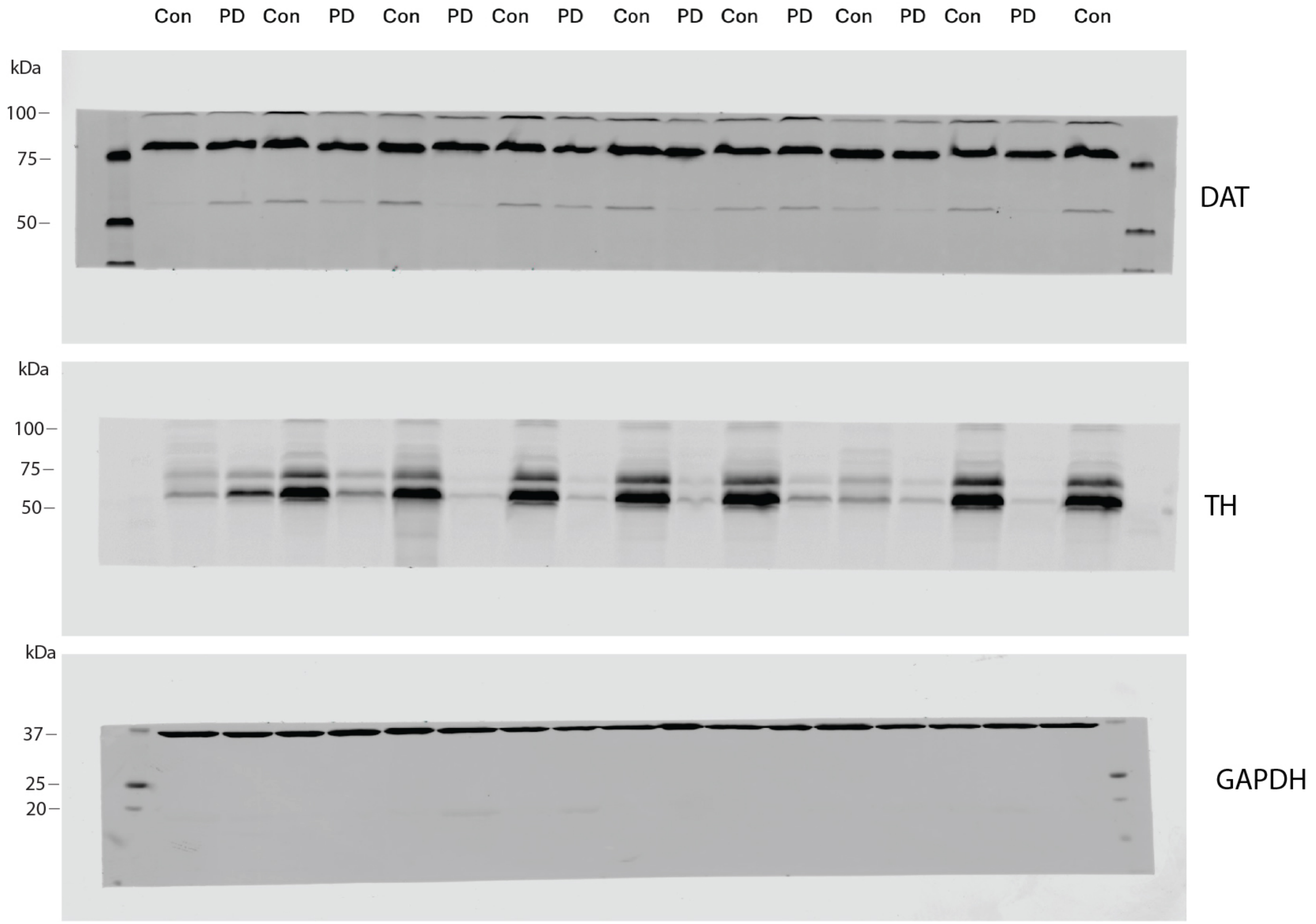

Supplemental Fig. 10: Uncropped Western blots corresponding to Figure 9 in main manuscript, showing dopamine transporter (DAT), tyrosine hydroxylase (TH), and GAPDH loading control in the substantia nigra of postmortem human tissue from neurologically intact controls (con) and Parkinson's disease (PD) brains.

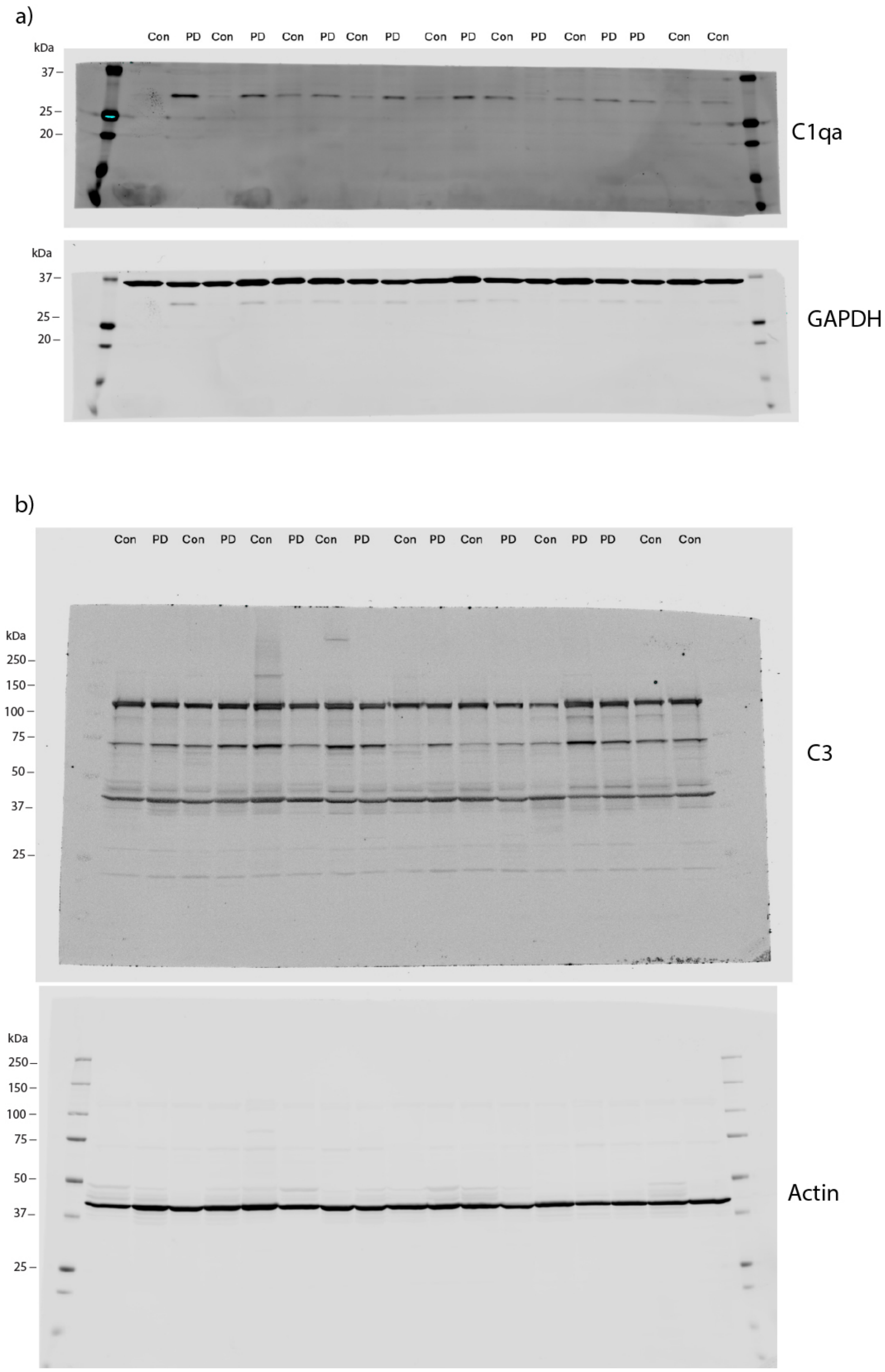

Supplemental Fig. 11: Uncropped Western blots corresponding to Figure 9 in main manuscript, showing C1qa (a), complement C3 (b), and associated loading controls in substantia nigra of postmortem human tissue from neurologically intact controls (con) and Parkinson's disease (PD) brains.

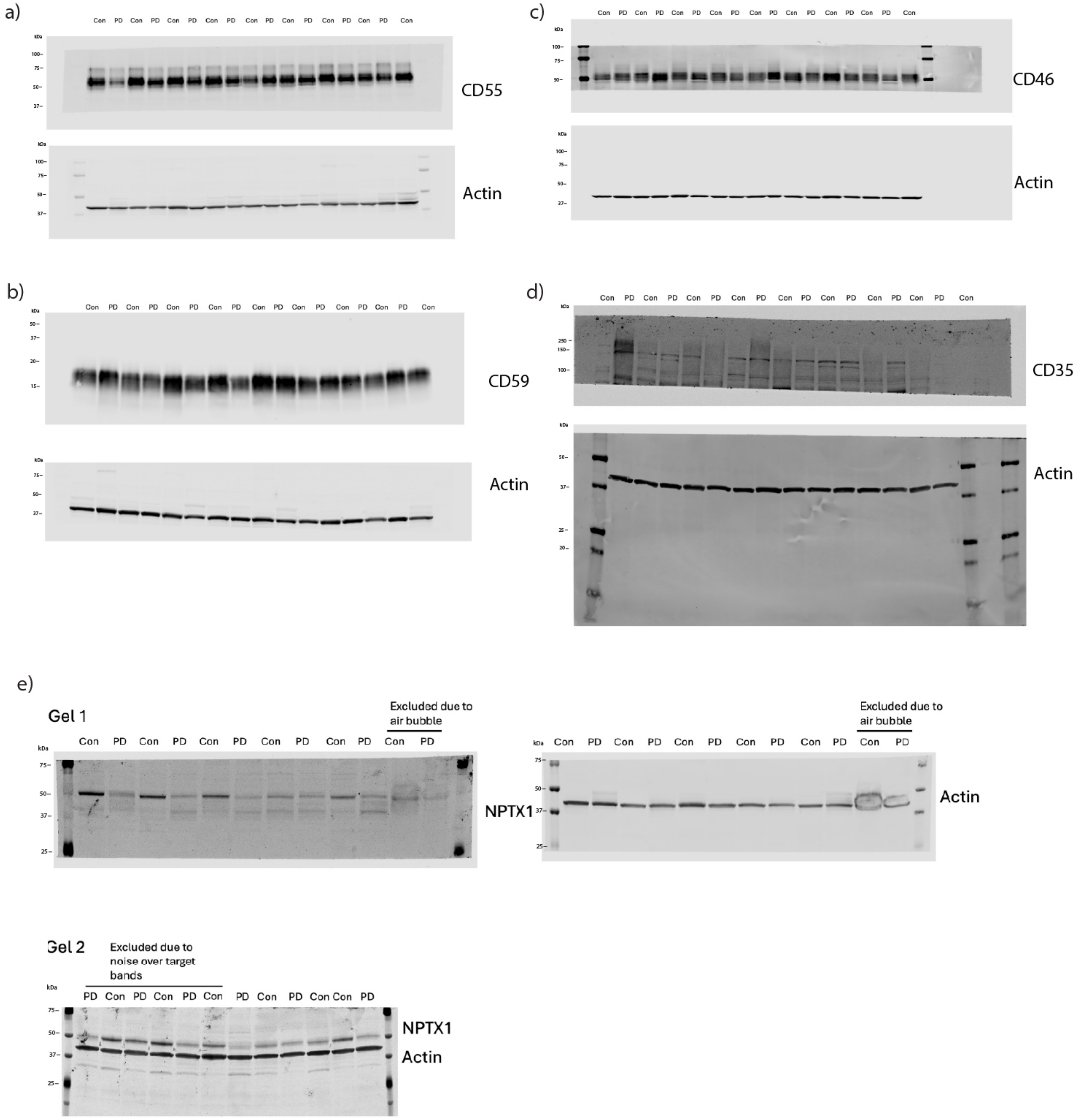

Supplemental Fig. 11: Uncropped Western blots corresponding to Figure 9 in main manuscript, showing CD55 (a), CD59 (b), CD46 (c), CD35 (d), and neuronal pentraxin 1 (NPTX1; e) and associated loading controls in substantia nigra of postmortem human tissue from neurologically intact controls (con) and Parkinson's disease (PD) brains.
